# Nanobodies targeting SARS-CoV-2 variants

**DOI:** 10.1016/j.apsb.2026.06.017

**Published:** 2026-06-12

**Authors:** Abhijeet Roy, Yang Yang, Lanying Du

**Affiliations:** a Institute for Biomedical Sciences, Georgia State University, Atlanta, GA 30303, USA; b Roy J. Carver Department of Biochemistry, Biophysics and Molecular Biology, Iowa State University, Ames, IA 50011, USA

**Keywords:** Coronavirus, COVID-19, SARS-CoV-2, Variants, Spike protein, Receptor-binding domain, Nanobodies

## Abstract

Severe acute respiratory syndrome coronavirus-2 (SARS-CoV-2), a beta-coronavirus, caused the recent global Coronavirus Disease 2019 (COVID-19) pandemic. Among the virus-encoded proteins, the surface spike (S) protein is critical for viral entry, membrane fusion, and pathogenesis, and its receptor-binding domain (RBD) initiates viral entry by binding to a cellular receptor. This makes the S an important therapeutic target for COVID-19. SARS-CoV-2 mutates frequently, giving rise to five major variants of concern, among which the Omicron variant and its subvariants are less sensitive to current therapeutic antibodies. The first part of this review describes the main protein constituents of SARS-CoV-2 and their functions, the S protein-mediated viral entry and fusion processes, and the main SARS-CoV-2 variants. Nanobodies are single-domain antibodies with high target-binding affinity, strong stability, and low production costs, whose small size facilitates their access to protein regions that are inaccessible to conventional antibodies. Thus, in the second part, we comprehensively review SARS-CoV-2-targeting nanobodies, including those that bind specifically to the RBDs of the S proteins, non-RBD S proteins, and non-S proteins of variants and subvariants of SARS-CoV-2, with the hope that this information will be valuable for the generation of novel SARS-CoV-2-targeting nanobodies with improved potency against COVID-19.

## Graphical abstract

This review provides an overview on nanobodies targeting SARS-CoV-2 variants, with the focus on those specific to the viral spike protein.

**Figure:**
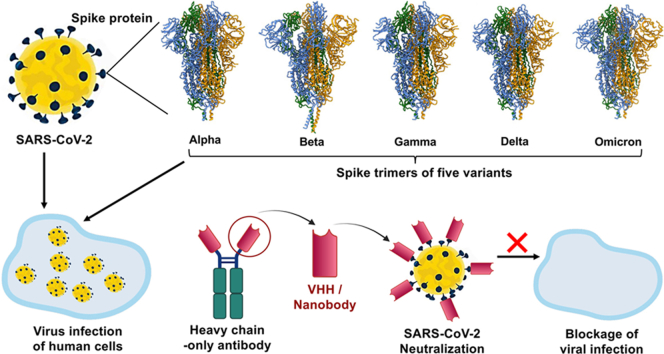

## 1 Introduction

Coronaviruses belong to the Coronaviridae family. Three highly emerging pathogenic coronaviruses have become critical public health problems. Severe acute respiratory syndrome coronavirus (SARS-CoV), which emerged in November 2002 and caused SARS outbreaks in 2003, spread worldwide with a mortality rate of 10%^1^^,^^2^. In June 2012, MERS-CoV, which caused Middle East respiratory syndrome coronavirus (MERS) outbreaks, emerged with a fatality rate of over 35%^3^^,^^4^. More recently, SARS-CoV-2 emerged in December 2019 and caused Coronavirus Disease 2019 (COVID-19), leading to a global pandemic that lasted for more than 4 years^5^^,^^6^. The number of infected individuals and the geographic spread of the COVID-19 epidemic have greatly outpaced those of SARS and MERS. According to the World Health Organization, at least 7.1 million deaths from COVID-19 among more than 779.2 million human cases worldwide have been reported as of May 31, 2026^7^.

The COVID-19 pandemic has had an unprecedented effect on the global economy and public health. This is largely due to the high rate of virus transmission, mutation, asymptomatic infection, and in some cases, inappropriate management strategies^8^, ^9^, ^10^. Virus transmission occurs through direct contact with an infected person or exposure to infected liquid droplets, surfaces, or items contaminated with SARS-CoV-2^11^. The most common symptoms, particularly during the early stage of virus infection, include fatigue and shortness of breath (38%), fever (87%), cough (67%), and loss of taste^12^. COVID-19 patients who have recovered are also continuing to be affected. Approximately one in eight patients who recover experience long-term COVID-19 symptoms^13^. COVID-19 can also deteriorate into pneumonia, multiple organ failure, and death^14^^,^^15^. Additionally, the virus may spread from humans to other animals, including ferrets, dogs, cats, mice, hamsters, and minks^16^.

During the evolution of SARS-CoV-2, clinical symptoms have shifted alongside the emergence of new variants, reflecting changes in viral transmissibility, tissue tropism, and immune evasion. As mentioned above, early in the COVID-19 pandemic (original strain and Alpha variant), virus infection often caused fever, cough, loss of taste or smell, and pneumonia, with a higher incidence of severe respiratory disease^17^. The Delta variant was associated with more severe illness, higher viral loads, and symptoms, such as fever, sore throat, cough, and shortness of breath, leading to increased hospitalizations^18^. With the emergence of Omicron and its subvariants, disease presentation became milder and more upper-respiratory–focused, commonly causing sore throat, nasal congestion, fatigue, headache, and cough, while loss of taste or smell became less frequent. Despite reduced severity, the high transmissibility of Omicron led to widespread infection, even among vaccinated individuals^19^. Overall, SARS-CoV-2 has evolved significantly from the original strain or early variants, with Omicron variants causing milder but more contagious infections, influenced by both viral mutations and increasing population immunity from vaccination and prior exposure.

## 2 SARS-CoV-2 major structural proteins and their functions

The degree of genomic similarity among human coronaviruses varies widely across species and genomic regions. SARS-CoV-2 shares the greatest sequence identity (about 79%–82%) with SARS-CoV, reflecting their close evolutionary relationship within the *Beta-coronavirus genus*^20^. In contrast, it shares much lower similarity with MERS-CoV (approximately 50%)^21^, and even less with seasonal human coronaviruses such as HKU1, NL63, OC43, and 229E, which belong to more divergent coronavirus lineages^22^. Conserved regions, particularly within the replicase (open reading frame (ORF1ab)) and structural protein genes, such as nucleocapsid (N) and envelope (E), show higher homology across coronaviruses. Conversely, the spike (S) protein, especially the receptor-binding domain (RBD), is more variable, accounting for differences in host range, receptor usage, and immune recognition^23^.

**Figure 1:**
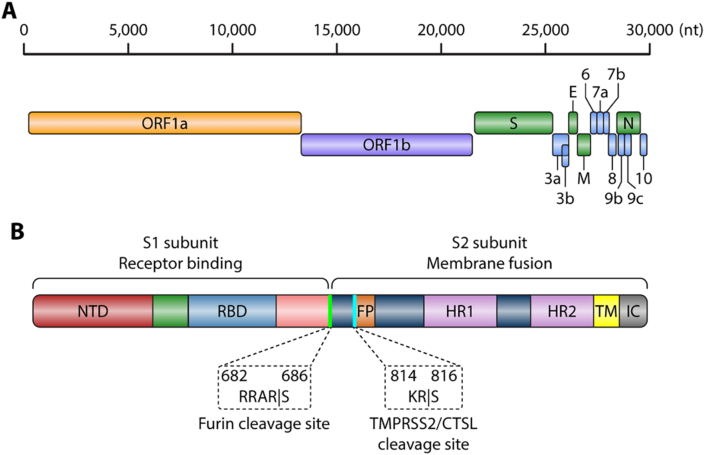
Genome organization of SARS-CoV-2 and domain structure of the viral spike (S) protein. (A) Schematics of SARS-CoV-2 genome organization. nt, nucleotide (*i*.*e*., bases). ORF, open reading frame; E, envelope; M, membrane; N, nucleocapsid. (B) Domain structure of SARS-CoV-2 S protein. NTD, N-terminal domain; RBD, receptor-binding domain; FP, fusion peptide; HR1 and HR2, heptad region 1 and 2; TM, transmembrane domain; IC, intracellular tail. The two protease cleavage sites on S protein are indicated by a green and cyan vertical line, respectively. The amino acid numbers and sequences of the two protease cleavage sites are shown. TMPRSS2/CTSL, transmembrane serine protease 2/cathepsin L.

SARS-CoV-2 is a single-stranded, positive-sense RNA virus with roughly 30 kilobases (kb) of nucleotides that encodes a large polyprotein of 9860 amino acids^24^. The genome of SARS-CoV-2 encodes 4 structural proteins, including S protein, membrane (M) protein, E protein, and N protein, 16 non-structural proteins (NSPs), and 9 small accessory proteins (Fig. 1A)^25^^,^^26^. Two large overlapping ORFs at the 5′ end, ORF1a and ORF1b, are transcribed by cellular ribosomes into polyproteins pp1a and pp1ab^26^. The polyproteins are subsequently processed by two viral proteases, papain-like protease (PL^pro^) and main protease (M^pro^ or 3CL^pro^), to generate the 16 NSPs, known as NSP1–NSP16^26^^,^^27^.

The S glycoprotein is critical for the life cycle of SARS-CoV-2. It boosts virulence and facilitates entry into host cells^28^. The S protein is a homotrimer, and each monomer consists of two functional subunits, S1 and S2, which mediate host receptor binding and membrane fusion, respectively^29^. The S1 subunit contains an N-terminal domain (NTD) and an RBD that attaches directly to the cellular receptor, angiotensin-converting enzyme 2 (ACE2), on host cells^30^. The highly variable RBD domain comprises a core region and an external subdomain called the receptor-binding motif (RBM), and the majority of binding sites between ACE2 and SARS-CoV-2 are located in the RBM region of the RBD (437–508)^30^^,^^31^. The S2 subunit is made up of a fusion peptide, two heptad repeats (HR1 and HR2) divided by a central helix, a transmembrane domain, and a cytoplasmic tail^32^. The S2 subunit controls membrane fusion between the virus and the host cell. The cleavage that occurs at the interface between the S1 and S2 subunits is known as the S1/S2 protease cleavage site^29^^,^^32^. To activate the proteins needed for virus fusion to the host cell membrane through irreversible conformational changes, host proteases cleave the S glycoprotein at the S1/S2 cleavage site. The S protein can exist in three different conformational states during the membrane fusion process: the native state (prefusion), the intermediate state (pre-hairpin), and the post-fusion hairpin state (stable)^33^^,^^34^. In addition, a significant amount of the S protein surface is *N-glycosylated*, which affects its conformation and dynamics^34^^,^^35^. The schematic structures of SARS-CoV-2 S protein, and its functional domains and related cleavage sites are shown in Fig. 1B.

The M protein is the primary component of the viral envelope. It is an O-linked glycoprotein with a molecular weight of ∼25–30 kDa. It promotes viral morphogenesis, virus assembly, and the recruitment of the S protein to the virus assembly and the prospective body^36^^,^^37^. The M protein is required for both genome packaging and nucleocapsid insertion into the virion. It has three main domains: an ectodomain at the N-terminus, three transmembrane helices, and an endodomain at the C-terminus. The M protein interacts with itself and other structural proteins, which is essential for membrane budding during the release of new virions^38^. Several antigenic epitopes have been identified in transmembrane regions of the M protein, and these have been targeted for the design of inhibitors and vaccines against SARS-CoV-2^39^.

The E protein is the smallest structural protein, with a molecular mass of 8–12 kDa. It is mostly located in the Golgi complex and endoplasmic reticulum of the host cell, where it is crucial for pathogenesis, virus assembly, and virus release^40^. It has three domains, including an N-terminal hydrophilic ectodomain, a hydrophobic transmembrane domain, and a hydrophilic C-terminal endodomain^40^. The E protein generates ion channels on the virus surface, which trigger the host inflammasome and loss of membrane potential. SARS-CoV-2 and SARS-CoV E proteins share about 95% sequence homology, with the M and E proteins being conserved in all *β*-coronaviruses^41^.

The N protein packages the viral RNA into a helical ribonucleoprotein and interacts with other structural proteins during virion assembly, resulting in genome encapsidation^42^. It consists of two highly conserved domains, the N-terminal RNA binding domain (N-NTD) and the C-terminal dimerization domain (N-CTD), which are characterized by three intrinsically disordered regions and a phosphorylated linker domain between the N-NTD and the N-CTD^43^. It is produced in large quantities during viral infection, and is highly immunogenic, making it an effective target for vaccine development. The N protein is also used for the diagnosis of SARS-CoV-2 infection because of its high sequence conservation, relatively low susceptibility to mutation, and strong protective immune response it induces in the host^42^.

## 3 SARS-CoV-2 life cycle and S protein-mediated viral entry and fusion

**Figure 2:**
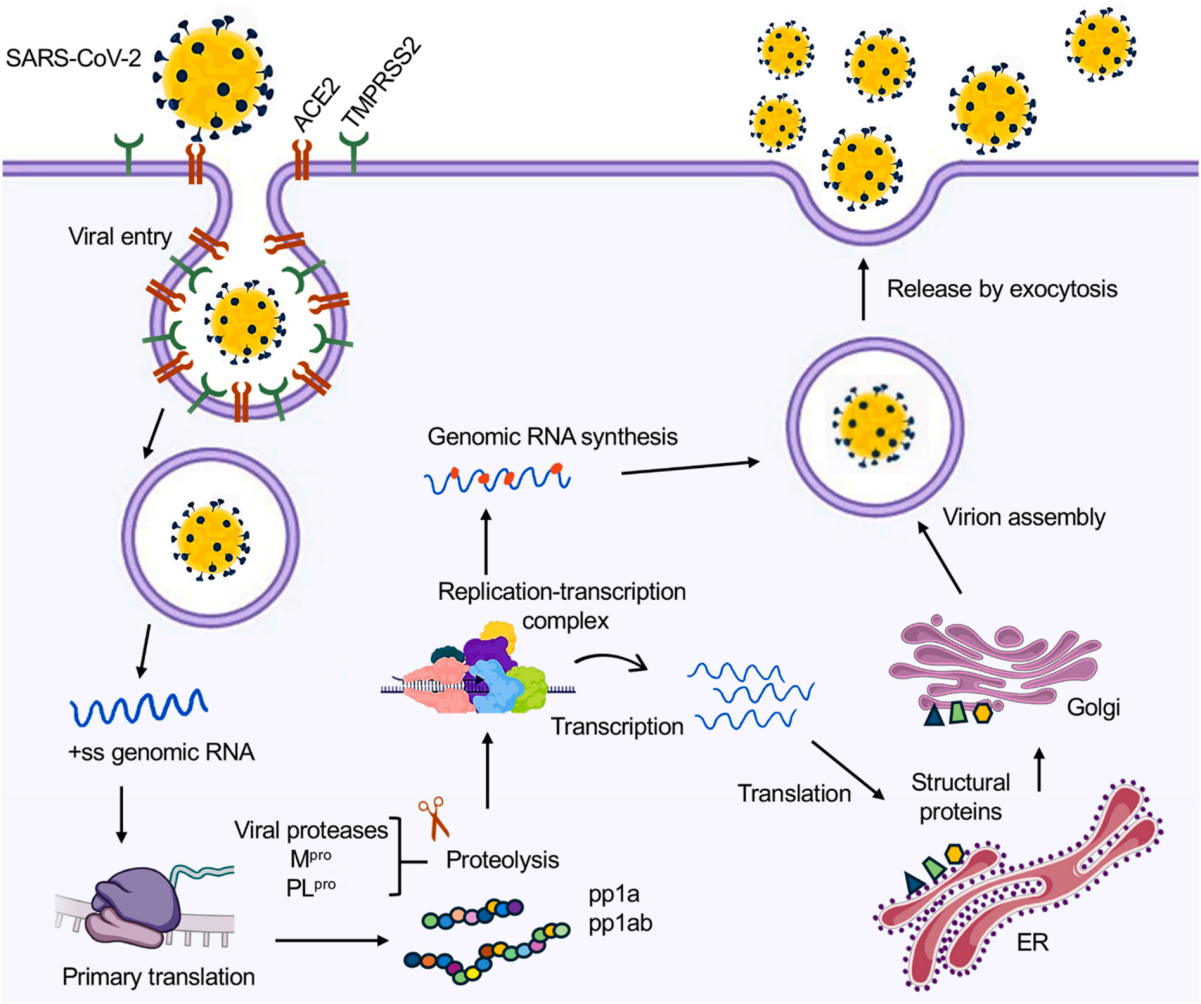
Life cycle of SARS-CoV-2. SARS-CoV-2 spike (S) protein interacts with angiotensin-converting enzyme 2 (ACE2) receptor and initiates the virus infection. Following receptor binding, the virus enters the target cell through proteolytic cleavage of the S protein by transmembrane serine protease 2 (TMPRSS2). Upon fusion of the viral and host cell membranes, the viral positive-sense, single-stranded RNA (+ssRNA) is released in the cytoplasm. The viral RNA initiates translation of polyproteins (pp1a/ab) by frameshifting mechanism. These polyproteins are subsequently cleaved into nonstructural proteins (NSPs) by the main protease (M^pro^) and papain-like protease (PL^pro^). Several NSPs interact with the RNA-dependent RNA polymerase to form the replicase–transcriptase complex responsible for the synthesis of full-length viral genome (replication) and sub-genomic RNAs (transcription). The viral structural proteins are expressed and translocated into the endoplasmic reticulum (ER). The nucleocapsid protein encapsidated genomic RNA and translocated with the structural proteins into the ER–Golgi intermediate compartment for virion assembly. The newly synthesized virions exit the cell membrane by exocytosis.

The life cycle of SARS-CoV-2 begins with host cell entry (Fig. 2). The S1 subunit, particularly its RBD, and the S2 subunit are responsible for S-mediated viral entry and fusion processes, making the S protein a primary target for the development of vaccines and therapeutic antibodies. Upon reaching the host cell surface, the RBD in the S1 subunit binds to the peptidase domain of the ACE2 receptor, resulting in a conformational change in the S1 subunit and the successive exposure of the S2′ proteolytic site in the S2 subunit^44^. The host transmembrane serine protease 2 (TMPRSS2) is highly expressed in lung epithelial cells and cleaves the S2 proteolytic site, which allows rapid cell entry of most SARS-CoV-2 variants^45^. However, the situation is different for Omicron variants of SARS-CoV-2, which primarily infect upper respiratory epithelial cells where TMPRSS2 expression is low. After attaching to cells, Omicron variants are slowly endocytosed into endo-lysosomes, where the S2′ proteolytic site is cleaved by cathepsin L^46^. Cleavage at the S2′ site further exposes the fusion peptide involved in fusion between the viral and host cellular membranes. Viral genomic RNA is released through the fusion pore into the host cell cytoplasm, where it hijacks the host protein synthesis machinery for the synthesis of viral polypeptides (pp1a and pp1ab), which are then cleaved by M^pro^ and PL^pro^ proteases^47^.

Viral RNA replication occurs in a replication-transcription complex containing RNA-dependent RNA polymerase. The N protein is translated into the cytoplasm, while other structural proteins, including S, M, and E proteins, are translated on the rough endoplasmic reticulum and undergo posttranslational modifications. The S protein is cleaved by furin or furin-like proprotein convertase at the S1–S2 junction in the Golgi apparatus, resulting in the non-covalent association of S1 and S2 subunits. Viral genomic RNA and structural proteins are transported to the endoplasmic reticulum–Golgi intermediate compartment for virion assembly. The single-stranded RNA is packaged internally with the N protein, while the S, E, and M proteins form the viral membrane. The newly formed virions are then released through exocytosis^48^.

## 4 SARS-CoV-2 variants

As SARS-CoV-2 continued to evolve, new variants exhibiting increased transmissibility and immune evasion emerged, which sparked a string of epidemiological waves of COVID-19 globally. Throughout the pandemic, the predominant variants of concern (VOCs) changed, with each new VOC exhibiting distinct characteristics and mutations, posing new public health challenges, because higher mutation loads increase the risk of vaccine escape or the blunting of efficacy of therapeutic agents and enhance infectivity, transmissibility, and/or severity to some degree (for the early VOCs, including Alpha, Beta, Gamma, and Delta, as well as for Omicron VOC).

The first VOC, the Alpha variant or lineage B.1.1.7, contains a remarkably high number of mutations within a single cluster, including four deletions, six synonymous mutations, and fourteen nonsynonymous mutations. Three different mutations in the S protein profoundly affect its properties. The first is in the RBM at position 501, where tyrosine replaces asparagine (N501Y), a residue involved in the RBD interaction with host ACE2. This increases the affinity of the virus for ACE2, making it easier for the virus to invade and cause infection^49^. The second is the P681H mutation, which is located near the furin cleavage site and the insertion sites of the four amino acids that connect the S1 and S2 subunits of the S protein^50^^,^^51^. This may increase S protein affinity for the ACE2 receptor and make it more susceptible to protease cleavage, thereby strengthening the virus's invasion of respiratory epithelial cells. The third is the deletion of amino acids 69 and 70, which may change the structure of the S protein, thus helping the virus overcome the host defenses^52^.

The Beta variant or lineage B.1.351 was originally identified in South Africa in December 2020 and thereafter expanded to other countries^53^. The severity of the disease was higher than that of other previous variants in South Africa because it was 50% more transmissible^54^^,^^55^. The risk of reinfection with this VOC was comparatively high because it was refractory to neutralizing antibodies in COVID-19 convalescent plasma^56^. Several S mutations are present in the Beta variant, including L18F, D80A, 242-244del, and R246I in the NTD, K417N, E484K, and N501Y in the RBD, D614G in S1, and A701V near the furin cleavage site of the S2 subunit^53^^,^^57^^,^^58^.

The Gamma variant, or lineage P.1, was discovered in Brazil in 2021. The transmissibility and viral load of the Gamma variant were up to 2-fold higher than those of previous variants, especially in younger patients^59^. This VOC was mutated at 10 sites in the S protein (L18F, T20N, P26S, D138Y, R190S, K417T, E484K, N501Y, H655Y, and T1027I), among which four sites are in the RBD (D614G, K417T, E484K, and N501Y), five sites in the NTD (L18F, T20N, P26S, D138Y, and R190S), and one site (H655Y) near the furin cleavage site in S2^60^^,^^61^. The N501Y, K417T, and/or E484K mutations, which are also found in Alpha and/or Beta VOCs, enable strong binding to human cells. The E484K mutation has been reported to aid antibody evasion^62^.

The Delta variant, or lineage B.1.617.2, was the fourth VOC to be identified^63^. It comprises about 23 mutations, which make it more transmissible and contagious^64^. There are two signature mutations in the S protein of the Delta variant, one at position 452 in the RBD region (L452R) and one at position 681 (P681R) in the furin cleavage site between S1 and S2, in addition to the primary D614G mutation, which was also shown in the other VOC strains. Similar to the other VOCs, the Delta variant has the T478K mutation, but it does not contain a substitution at residue 484^65^. The most prominent mutations observed in the S protein include T19R, L452R, T478K, D614G, and D950N, as well as two deletions at positions 157 and 158, which help this variant evade the immune system^64^. The L452R mutation increases the binding affinity of this VOC for the ACE2 receptor^64^^,^^65^.

A highly transmissible VOC, Omicron or lineage B.1.1.529, was discovered in South Africa in November 2021^66^. More than 60 mutations have been identified in this variant, including deletions, insertions, and substitutions, and almost half of them occur in the S protein^67^. Several mutations, including a D69–70 deletion, T95I, G142D/D143–145 deletion, K417N, T478K, N501Y, N655Y, N679K, and P681H, are common to other VOCs^68^. In contrast to the RBD of the Delta variant, which has two mutations (L452R and T478K), the RBD of the Omicron variant has about 15 mutations that impact virus transmissibility, infectivity rate, viral entry, and host immunity, making them the most concerning mutations^69^.

A number of Omicron subvariants that emerged over a relatively short period of time have been identified, which include BA.1, BA.1.1, BA.2, BA.4, BA.5, BQ.1, BQ.1.1, LB.1, and XBB.1.5, as well as JN.1, KP.2, KP.3, XEC, NB.1.8.1, LP.8.1, and XFG^70^^,^^71^. These subvariants have genetic similarities to the Omicron variant and are associated with increased transmissibility. Each subvariant has distinct traits and mutations^70^. The original Omicron variant, known as BA.1 (B.1.1.529.1), carries 30 substitutions, six deletions, and three insertions in the S protein, including 13 unique mutations^72^, such as S371L, G446S, and G496S in the RBD, that are not found in other variants^73^. BA.1.1 has a unique mutation (R346K) that enhances its binding to ACE2. Both BA.1 and BA.1.1 result in less severe symptoms, but are more contagious than previous variants, and show significant immune evasion^74^. BA.2 shares 32 mutations with BA.1, including 19 unique mutations mostly located in the S protein, and is about 1.4-fold more contagious than BA.1^75^^,^^76^. BA.4 and BA.5, which have specific mutations (L452R and F486V) in the S protein^77^, contribute to the faster spread relative to previous variants and effective evasion of host immune defenses. XBB.1.5 has a unique mutation (F486P) in the S protein, which increases its effective binding to ACE2^78^. JN.1 has been classified as a variant of interest because of its potential to evade immunity. While the transmissibility of JN.1 appears to be higher than that of previous variants, it is not yet clear if this Omicron subvariant causes more severe disease^79^. The KP.3 subvariant is a descendant of Omicron BA.5 and XBB.1.5 sub-lineages^80^. XEC is a recombinant strain of KP.3.3 and KS.1.11, but there is no evidence that its symptoms differ or are more severe than those of other recent subvariants^81^.

**Figure 3:**
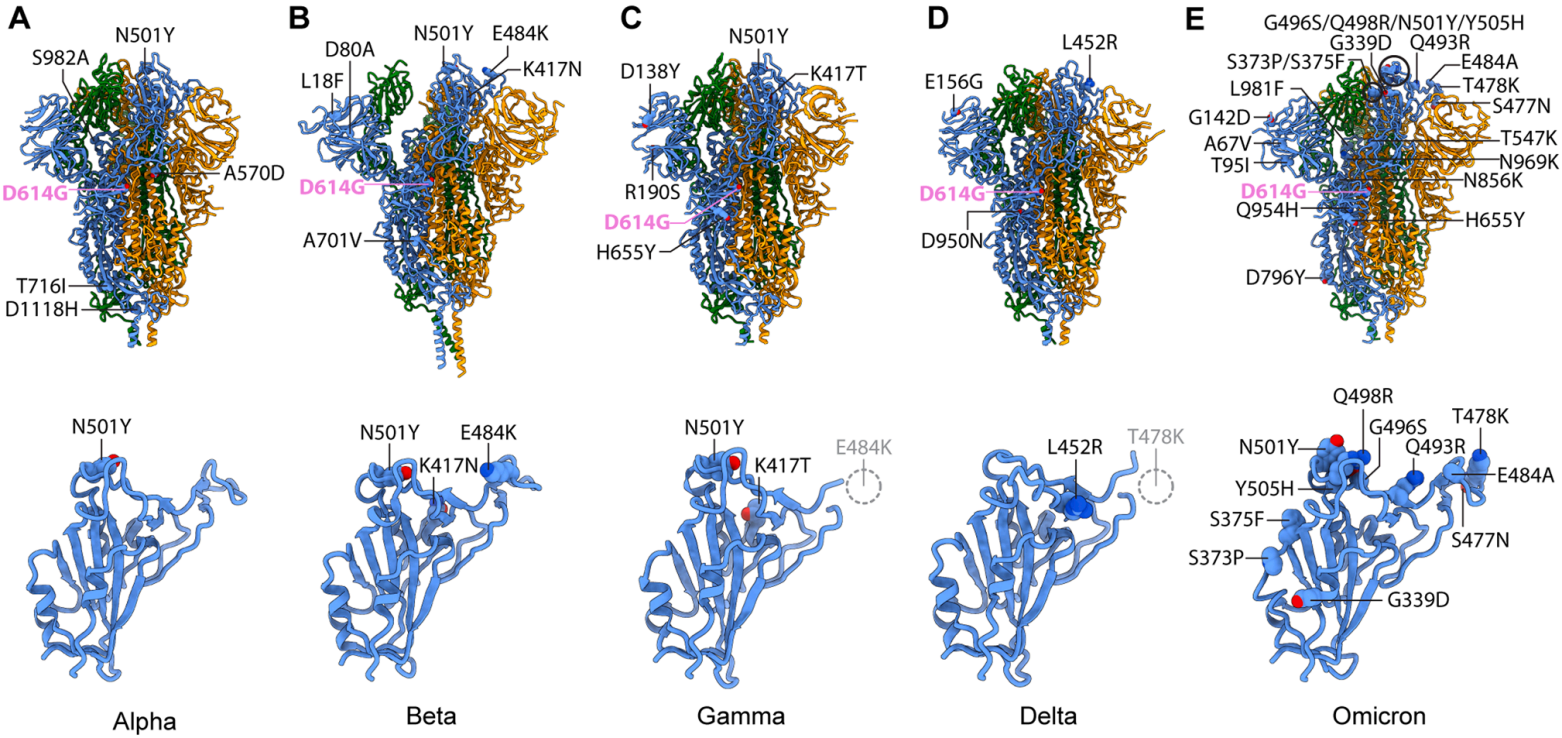
Cryo-EM structures of the spike (S) proteins from different SARS-CoV-2 variants of concern (VOCs). (A) Structure of SARS-CoV-2 Alpha variant S protein in the closed state (PDB 9CRE). The overall structure of the S trimer is shown in the upper panel, and a zoomed-in view of the RBD from one S subunit is shown in the lower panel. Mutations compared with the wild-type S are indicated. (B) Structure of SARS-CoV-2 Beta variant S protein in the transition state (PDB 7WEV). (C) Structure of SARS-CoV-2 Gamma variant S protein trimer in the closed state (PDB 9CRG). K484 of RBD, which is located in a loop not modeled in the structure, is indicated by a gray-dashed circle. (D) Structure of SARS-CoV-2 Delta variant S protein trimer in the closed state (PDB 9CRh). K478 of RBD, which is located in a loop not modeled in the structure, is indicated by a gray-dashed circle. (E) Structure of SARS-CoV-2 Omicron variant S protein trimer in the closed state (PDB 7WK2). D614G mutation, which is shared by all variants shown here, is highlighted in magenta.

The cryo-EM structures of the S proteins of the above five SARS-CoV-2 VOCs and relevant critical mutations are illustrated in Fig. 3A–E. All the S proteins of these VOCs have a trimeric structure. As described earlier and shown in these figures, several key mutations in the S protein, such as L18F, T478K, E484K, N501Y, D614G, H655Y, and P681H, are common to two or more VOCs, whereas other S-protein mutations, including R346K, S371L, K417N/T, G446S, L452R, F486P, F486V, G496S, P681R, and D950N, are unique to particular VOCs or Omicron subvariants. Overall, a series of these mutations, such as R346K, K417T, G446S, L452R, T478K, E484K, F486P/F486V, G496S, and N501Y, play important roles in enhancing viral binding affinity to the ACE2 receptor or destabilizing ACE2, affecting antibody binding or the conformation of the RBD, or increasing immune evasion and/or antibody escape^62^^,^^82^, ^83^, ^84^, ^85^, ^86^. Other mutations, such as L452R, D614G, P681H, P681R, and D950N, may increase virus infectivity, enhance virus fitness, or improve S protein cleavage and membrane fusion with increased transmissibility, thereby escalating viral pathogenesis in a variety of reports^83^^,^^87^, ^88^, ^89^, ^90^, ^91^.

## 5 Nanobodies and related functions

**Figure 4:**
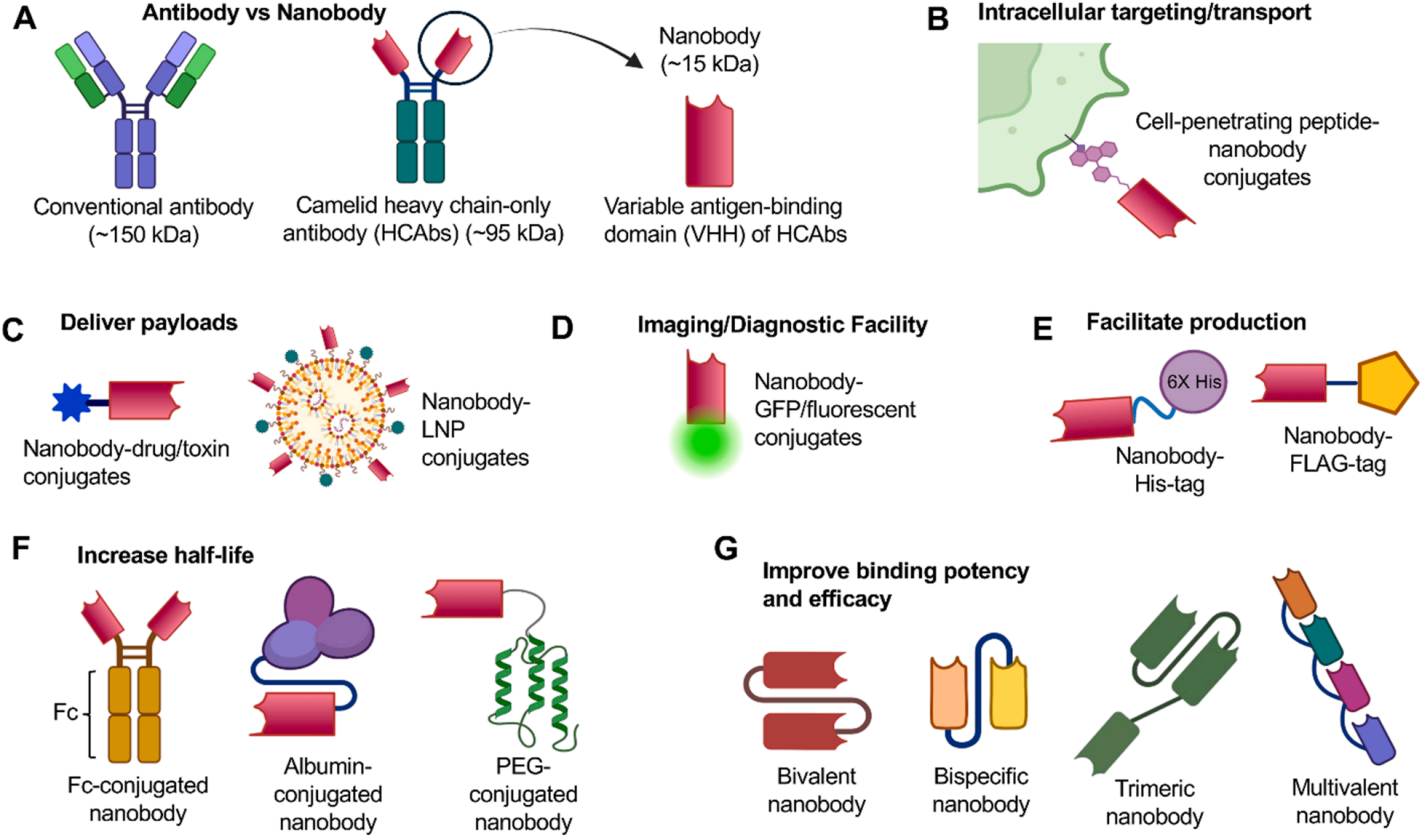
Schematic overview of nanobody structure and engineering modifications to enhance pharmacokinetics, potency, and other functionalities. (A) Structural comparison of a nanobody and a conventional antibody. Strategies of nanobody engineering to conjugate it with cell-penetrating peptides (B), drugs or lipid nanoparticle (LNP) (C), green fluorescent protein (GFP) or other fluorescent proteins (D), His_6_ or other production/purification tags (E), as well as Fc and other fusion proteins (F) for promoting nanobody delivery, production, and purification, increasing nanobody's half-life, or using nanobody as imaging or diagnostic supplies. (G) Nanobodies can be engineered in different formats (bivalent, bispecific, trimeric, or multivalent) to improve their binding potency and efficacy.

Nanobodies are derived from heavy-chain-only antibodies (HCAbs). In contrast to conventional (traditional) antibodies, which have a molecular mass of ∼150 kDa with two heavy chains and two light chains, camelid HCAbs consist of an antigen-binding region (*i*.*e*., a single variable heavy chain domain (VHH or nanobody: 15 kDa)) that is directly connected to an Fc region consisting of CH2 and CH3 constant domains (Fig. 4A)^92^. Nanobodies, or VHHs, are composed of three complementarity-determining regions (*i*.*e*., CDR1, CDR2, and CDR3) and four framework regions (FR1, FR2, FR3, and FR4)^93^. Nanobodies are functionally comparable to the fragment antigen-binding region (Fab region) of an IgG antibody, but they exhibit improved solubility, stability, and other characteristics described below. Altogether, nanobodies offer a wider range of advantages relative to conventional antibodies, particularly in their biophysical properties, productivity, and therapeutic potential, as illustrated below.

First, because of their elongated antigen-binding component (*i*.*e*., CDR3 domain), nanobodies can access regions that are normally inaccessible to conventional antibodies^94^. Likewise, the amino acid residues of the CDR3 region are more variable than those of conventional antibodies, and they can recognize a greater number of epitopes, some of which are “hidden” or cryptic^95^. Additionally, the small size and single-domain structure of nanobodies allow them to penetrate dense tissues far more effectively than full-length IgG antibodies or their functional fragments. Moreover, nanobodies could conjugate with cell-penetrating peptides for enhanced delivery of therapeutics or other agents into cells (Fig. 4B)^96^. The above properties of nanobodies would enable the development of nanobody-drug/tracer conjugates that can diffuse into tissues and tumors or reach unusual epitopes where traditional antibody-drug conjugates cannot (Fig. 4C). Furthermore, nanobodies have high antigen-binding affinity with equilibrium dissociation constants within the nanomolar to picomolar range, despite having a smaller antigen-binding region^97^. This function makes it possible for nanobodies to achieve more targeted and effective drug delivery and better tissue imaging when conjugated to fluorescent proteins (Fig. 4D)^98^^,^^99^. Second, nanobodies exhibit exceptional stability, solubility, and ease of production in microbial systems, making them cost-effective and less prone to aggregation. Unlike traditional antibodies, nanobodies retain most of their antigen-binding activity when exposed to chemical denaturants or proteases, non-physiological pH levels (pH 3.0–9.0), and high temperatures up to 80 °C^100^. Nanobodies can be efficiently selected from diverse libraries (immune, naive, and synthetic) using a variety of selection techniques, such as phage display, ribosome display, and yeast surface display, and they are easily expressed in various prokaryotic, eukaryotic, and plant expression systems, in the presence or absence of a facilitating tag such as His_6_ or FLAG, with a high yield and maintaining their properties (Fig. 4E)^101^. Overall, nanobodies offer unique advantages and high engineering flexibility, but require modifications if needed (as described below), to fully match the pharmacokinetic and immunological strengths of conventional antibodies.

Third, nanobodies exhibit great preclinical and clinical therapeutic potentials by targeting a variety of viral pathogens, cancers, and other diseases through different mechanisms of action^102^, ^103^, ^104^, ^105^. For example, neutralizing nanobodies prevent viral infection by binding to critical viral structures in a way that blocks the virus from entering or replicating inside host cells^106^. They may function by attaching to viral surface proteins (*e*.*g*., SARS-CoV-2 S or RBD) and physically preventing the virus from binding to its host receptor (*e*.*g*., ACE2)^106^. Nanobodies can also stabilize viral proteins in a non-functional conformation, stopping membrane fusion and cell entry^105^. Additionally, nanobodies create a physical block around entry-related surfaces, limiting receptor engagement and co-receptor interaction^103^. Furthermore, nanobodies can modulate immunity by targeting cytokine signaling molecules and guiding them to specific cells^107^. Indeed, a series of nanobodies have advanced into clinical trials, or have been approved for use in humans to treat non-viral diseases, such as cancers, acquired thrombotic thrombocytopenic purpura, asthma, inflammation, and rheumatoid arthritis^108^, ^109^, ^110^, ^111^, ^112^, ^113^, ^114^. The successful clinical applications and approvals of nanobodies against non-viral human diseases bring significant hope for using nanobodies to treat virus-caused human diseases, such as SARS-CoV-2-caused COVID-19.

Compared to conventional antibodies, the unbound nanobodies with the low molecular weight could be rapidly eliminated from the bloodstream through renal clearance, which has a threshold of around 60 kDa^92^. Therefore, nanobodies generally present a short *in-vivo* half-life and lack of Fc-mediated effector functions, which would limit their natural immune engagement. Nevertheless, these shortcomings can be adequately addressed by genetically engineering nanobodies with the Fc region of human IgG or IgM, or other fusion tags, such as an albumin-binding domain or related nanobodies, as well as conjugating to polyethylene glycol (PEG), known as PEGylation, which would extensively extend the half-life of target nanobodies (Fig. 4F)^115^, ^116^, ^117^, ^118^, ^119^, ^120^. These fusion-tagged nanobodies have shown improved potency with effective therapeutic potentials against viral and non-viral pathogens^115^^,^^118^^,^^119^^,^^121^. In addition, the binding avidity, neutralization efficiency, and/or protective effect of nanobodies can also be increased when they are fused to two or more homologous or heterologous molecules to generate dimeric (bivalent, bispecific, or biparatopic), trimeric, or multimeric forms (Fig. 4G)^94^. Notably, these nanobody derivatives have excellent functionality and can be effectively produced in large quantities in *E*. *coli* or yeast expression systems, after which they remain soluble, enabling large-scale production^94^^,^^95^.

## 6 Nanobodies targeting SARS-CoV-2 variants

### 6.1 Nanobodies targeting the RBD of SARS-CoV-2 early variants

Nanobodies are promising therapeutic tools for the treatment of diseases caused by respiratory pathogens, such as coronaviruses, respiratory syncytial virus, and influenza viruses. Coronavirus infection is generally limited to the nasopharynx and lungs, where only 0.2% of traditional antibodies can penetrate after intravenous injection, so their clinical impact is mild^122^. By contrast, nanobodies can be delivered *via* pulmonary administration due to their unique biological and biophysical properties. They are also tolerant of harsh manufacturing conditions. Their small size enables direct delivery of nanobodies into the lungs using a nebulizer^123^.

The RBD of SARS-CoV-2 S protein is considered the most prominent target for the development of nanobody-based therapies. Most nanobodies that targeted early variants (*i*.*e*., Alpha, Beta, Gamma, and Delta) of SARS-CoV-2 were generated from immunized animals (alpaca, camels, llama, sharks, or mice) or synthetic nanobody libraries and showed efficacy by directly or indirectly engaging epitopes in the RBD. Therefore, these RBD-targeting nanobodies mainly function by blocking viral entry into host cells. The RBD-targeting nanobodies and those targeting other S regions or non-S proteins of SARS-CoV-2 variants are summarized in Table 1^124^, ^125^, ^126^, ^127^, ^128^, ^129^, ^130^, ^131^, ^132^, ^133^, ^134^, ^135^, ^136^, ^137^, ^138^, ^139^, ^140^, ^141^, ^142^, ^143^, ^144^, ^145^, ^146^, ^147^, ^148^, ^149^ and described below.

**Table 1:** Representative nanobodies targeting SARS-CoV-2 variants.

Nanobody	Screening method	Antigen/immunization	Target protein	Binding affinity to SARS-CoV-2 (*K*_D_ values) aMethod/instrument	SARS-CoV-2 neutralization (*in vitro*: IC_50_ values)		SARS-CoV-2 VOCs	*In vivo* protection	Ref.
					Pseudovirus	Live virus			
**Nanobodies targeting RBD of SARS-CoV-2 early variants**									
saRBD-1	Phage display	SARS-CoV-2 S-RBD (Alpaca)	RBD	0.67 nmol/L (WT S) 1.88 nmol/L (WT S1) 0.75 nmol/L (WT RBD) a BLI	4.26 nmol/L (SARS-CoV-2 WT)	5.82 nmol/L (WA1-WT) 15.84 nmol/L (Alpha) 19.95 nmol/L (Beta) 19.87 nmol/L (Gamma) 16.43 nmol/L (Delta)	Alpha Beta Gamma Delta	ND	124
7A3	Phage display	SARS-CoV-2 RBD (Dromedary camel)	RBD S2	0.2 nmol/L (WT RBD) 0.23 nmol/L (Alpha RBD) 0.26 nmol/L (Beta RBD) 0.8 nmol/L (Gamma RBD) 0.42 nmol/L (Delta RBD) a Octet RED96 system (ForteBio)	3 nmol/L (WT) 0.6 nmol/L (Alpha) 1 nmol/L (Beta) 2 nmol/L (Gamma)	N/S	Alpha Beta Gamma	Protects hACE2-Tg mice from SARS-CoV-2 Beta/Delta variant infection	125
2F4	Phage display	SARS-CoV-2 RBD (Llama)	RBD	3.87 × 10^−10^ mol/L (WT RBD) a BLI	3.52 nmol/L (WT) 3.58 nmol/L (Delta)	ND	Delta	Fc format protects hamsters from SARS-CoV-2 (human/RUS/Nsk-FRCFTM-1/2020) infection	130
ShAb01	Phage display	SARS-CoV-2 RBD RBD-ferritin S-ferritin (Sharks)	RBD	47.1 nmol/L (WA-1-WT RBD) 53.9 nmol/L (Alpha RBD) 70.8 nmol/L (Beta RBD) 38.9 nmol/L (Delta RBD) a BLI	609 ng/mL (WA-1-WT) 420 ng/mL (Alpha) 619 ng/mL (Beta) 188 ng/mL (Delta)	ND	Alpha Beta Delta	Protects K18-hACE2 mice from SARS-CoV-2 WA-1/2020 strain infection	131
ShAb02	Phage display	SARS-CoV-2 RBD RBD-ferritin S-ferritin (Sharks)	RBD	85.7 nmol/L (WA-1-WT RBD) 72.4 nmol/L (Alpha RBD) 71.6 nmol/L (Beta RBD) 14.9 nmol/L (Delta RBD) a BLI	52 ng/mL (WA-1-WT) <23 ng/mL (Alpha) <23 ng/mL (Beta) 15 ng/mL (Delta)	ND	Alpha Beta Delta	Protects K18-hACE2 mice from SARS-CoV-2 WA-1/2020 strain infection	131
Sb#68	Synthetic nanobody library	Synthetic	RBD	9 nmol/L (WT RBD) a Grating-coupled interferometry	138 nmol/L (WT) 299 nmol/L (Alpha) 84 nmol/L (Beta) 90 nmol/L (Delta)	377 nmol/L (WT)	Alpha Beta Delta	ND	132
RBD-1-2G	Synthetic humanized nanobody library	Synthetic	RBD	14.3 nmol/L (WT RBD) a BLI	511.6 nmol/L (WT) 746.5 nmol/L (Alpha)	1211 nmol/L (WT)	Alpha	ND	133
Nb12	Phage display	SARS-CoV-2 S (Camelid nanomice)	RBD	3 × 10^−8^ mol/L (WA1-WT RBD) a BLI	7145 pmol/L (WA-1-WT) 6910 pmol/L (R683G mutation) 6575 pmol/L (K417N mutation) 10048 pmol/L (E484K mutation) 5912 pmol/L (N501Y mutation)	ND	Alpha Beta Gamma	ND	134
Nb30	Phage display	SARS-CoV-2 S (Camelid nanomice)	RBD	6.55 × 10^−9^ mol/L (WA1-WT RBD) a BLI	3705 pmol/L (WA-1-WT) 3837 pmol/L (R683G mutation) 2607 pmol/L (K417N mutation) 7358 pmol/L (E484K mutation) 3677 nmol/L (N501Y mutation)	ND	Alpha Beta Gamma	ND	134
**Nanobodies targeting RBD of SARS-CoV-2 Omicron and its subvariants**									
Nanosota-9	Phage display	S Ectodomain of Omicron BA.5 (Alpaca)	RBD	0.078 nmol/L (BA.5) 0.06 nmol/L (XBB.1.5) 29.4 nmol/L (JN.1) a SPR	Fc format: 8 ng/mL (BA.5) 15 ng/mL (XBB.1.5) 9 ng/mL (JN.1)	Fc format: 10 ng/mL (BA.5) 98 ng/mL (XBB.1.5) 44 ng/mL (JN.1)	BA.5 XBB.1.5 JN.1	Fc format protects C57BL/6J mice from SARS-CoV-2 Omicron BA.5, XBB.1.5, and JN.1 infection	135
A14	Phage display	SARS-CoV-2 S1 and RBD (Alpaca)	RBD	0.065 nmol/L (BA.1) a SPR	5.49 nmol/L (WT) 5.57 nmol/L (BA.1) 18.58 nmol/L (BA.4/5) 20.69 nmol/L (BQ.1.1) 13.59 nmol/L (XBB.1)	ND	BA.1 BA.4/5 BQ.1.1 XBB.1	ND	136
H6	Phage display	SARS-CoV-2 WT /Beta S (Llama)	RBD	22.7 nmol/L (BA.1) 7.08 nmol/L (BA.4/5) a BLI	ND	Trimeric format: 0.219 nmol/L (BA.5) 1.32 nmol/L (XBB.1.5)	BA.5 XBB.1.5	Trimeric format protects hamsters from SARS-CoV-2 Omicron BA.5 infection	137
B5-5	Phage display	SARS-CoV-2 WT /Beta S (Llama)	RBD	0.355 nmol/L (BA.1) 0.684 nmol/L (BA.4/5) a BLI	ND	Trimeric format: 352 nmol/L (BA.5) 262 nmol/L (XBB.1.5)	BA.5 XBB.1.5	Trimeric format protects hamsters from SARS-CoV-2 Omicron BA.5 infection	137
Nb1	Phage display	SARS-CoV-2 S1 (Alpaca)	RBD	Nb1-Fc 3.58 × 10^−10^ mol/L (XBB.1.16) a SPR	Nb1-Fc: 8.70 ng/mL (WT) 6.50 ng/mL (BA.1) 25.40 ng/mL (BA.2) 73.15 ng/mL (BA.3) 26.78 (BA.5) 25.86 ng/mL (BA.2.75) 30.86 ng/mL (BF.7) 320.2 ng/mL (BQ.1) 7.69 ng/mL (EG.5.1) 2.82 ng/mL (XBB.1.5) 47.42 ng/mL (JN.1)	Nb1-Fc: 23.57 ng/mL (WT) 121.1 ng/mL (BA.5) 179.5 ng/mL (BQ.1) 169 ng/mL (EG.5.1)	BA.1 BA.2 BA.3 BA.5 BA.2.75 BF.7 BQ.1 EG.5.1 XBB.1.5 JN.1	ND	138
Re32D03	Phage display	SARS-CoV-2 WT & mutant S (Alpaca)	RBD	350 pmol/L (BA.2.75) 20000 pmol/L (BA.5) 50000 pmol/L (BQ.1) a BLI	IC_99_: 2 nmol/L (BA.2.75)	IC_99_: 167 pmol/L (WT) 167 pmol/L (Alpha) 50 pmol/L (Delta)	BA.2.75	Re32D03 protects hamsters from SARS-CoV-2 WT infection	140
Nb4	Synthetic library	Synthetic	RBD	7.4 nmol/L (BA.1) a BLI	ND	1.5 μg/mL (BA.1.1) 20 μg/mL (BA.2.3)	BA.1.1 BA.2.3 BA.5	Multivalent format (Nb4-16t) protects BALB/c mice from Omicron BA.1.1, BA.2.3, and BA.5 infection	141
Tnb04-1	Yeast display	SARS-CoV-2 BA.4/5 S (Alpaca)	RBD	6.48 × 10^−10^ mol/L (WT RBD) a SPR	0.012 μg/mL (WT) 0.010 μg/mL (Delta) 0.007 μg/mL (BA.1) 0.016 μg/mL (BA.2) 0.030 μg/mL (BA.4/5) 0.023 μg/mL (BF.7) 0.029 μg/mL (BQ.1.1) 0.016 μg/mL (XBB) 0.011 μg/mL (XBB.1.5) 0.011 μg/mL (EG.5.1) 0.027 μg/mL (HK.3) 0.028 μg/mL (HV.1) 0.025 μg/mL (JD.1.1) 0.016 μg/mL (JN.1)	0.025 μg/mL (Delta) <0.2 μg/mL (BA.1) <0.2 μg/mL (BA.2) <0.2 μg/mL (BA.5.2) <0.2 μg/mL (BQ.1.1) <0.2 μg/mL (XBB) >0.2 μg/mL (XBB.1.5) <0.2 μg/mL (EG.5.1)	BA.1 BA.2 BA.4/5 BF.7 BQ.1.1 XBB XBB.1.5 EG.5.1 HK.3 HV.1 JD.1.1 JN.1	Protects hamsters from SARS-CoV-2 Omicron XBB.1.5 infection	142
9A7	Phage display	Sequential immunization with different RBD variants (Llama)	RBD	1.57 × 10^−10^ mol/L (BA.1 RBD)	3.3 nmol/L (WT)	ND	BA.5 BQ.1.1 XBB.1	ND	130
				4.07 × 10^−10^ mol/L (BA.5 RBD)	2.7 nmol/L (BA.5)				
				2.39 × 10^−9^ mol/L (XBB.1.5 RBD)	14.1 nmol/L (BQ.1.1)				
				a BLI	11.4 nmol/L (XBB.1)				
**Nanobodies targeting non-RBD S protein of SARS-CoV-2 variants**									
N235	Phage display	SARS-CoV-2 S and NTD (Alpaca)	NTD	0.04 nmol/L (Alpha) 0.05 nmol/L (Beta) 0.02 nmol/L (Gamma) 0.08 nmol/L (Delta) 0.05 nmol/L (Lambda) 0.03 nmol/L (BA.1) 0.03 nmol/L (BA.3) 0.01 nmol/L (BA.5) 0.04 nmol/L (XBB.1.5) 0.05 nmol/L (XBB.1.16) a SPR	4.5 μg/mL (WT) 10.3 μg/mL (Delta) 5.5 μg/mL (BA.1) 7.2 μg/mL (BA.1.1) 1.1 μg/mL (BA.2) 2.0 μg/mL (BA.2.12.1) 3.0 μg/mL (BA.2.75) 0.3 μg/mL (BA.3) 2.2 μg/mL (BA.4/5) 1.4 μg/mL (BQ.1.1) 1.9 μg/mL (BF.7) 2.0 μg/mL (XBB) 3.0 μg/mL (XBB.1.5) 2.1 μg/mL (EG.5.1) 0.8 μg/mL (JN.1)	IgM-conjugated format (NM235): 0.096 μg/mL (Delta) 0.031 μg/mL (BA.1) 0.012 μg/mL (BA.1.1) 0.015 μg/mL (BA.2) 0.328 μg/mL (BA.4) 0.592 μg/mL (BA.5) 0.134 μg/mL (BF.7) 0.935 μg/mL (XBB) 0.91 μg/mL (EG.5.1)	Delta	N235 and IgM-conjugated format (NM235) protects hACE2-Tg mice from SARS-CoV-2 Omicron XBB and BA.1 infection	143
							BA.1		
							BA.2		
							BA.2.12.1		
							BA.2.75		
							BA.3		
							BA.4/5		
							BQ.1.1		
							BF.7		
							XBB.1.5		
							XBB.1.16		
							BA.2.86		
							EG.5.1		
							JN.1		
SR01	Phage display	SARS-CoV-2 (WT) S (Llama)	NTD	0.56 nmol/L (WT) 0.59 nmol/L (Alpha) 0.2 nmol/L (Beta) a SPR	188 nmol/L (WT)	Fc format: 9.42 nmol/L (WT) 3.77 nmol/L (Alpha) 70.3 nmol/L (Beta) 6.64 nmol/L (Omicron)	Alpha Beta Omicron	Fc format protects hamsters from SARS-CoV-2 -WT infection	144
S102	Phage display	SARS-CoV-2 S (Alpaca)	S2	19.6 pmol/L (WT) 28.4 pmol/L (BA.1) 18.8 pmol/L (BA.2) a Octet RED96 system (ForteBio)	1.45 μg/mL (WT) 5.68 μg/mL (Alpha) 2.49 μg/mL (Beta) 0.37 μg/mL (Delta) 0.59 μg/mL (BA.1) 0.54 μg/mL (BA.1.1) 0.63 μg/mL (BA.2) 0.53 μg/mL (BA.4) 0.53 μg/mL (BA.5) 0.95 μg/mL (BF.7) 0.91 μg/mL (XBB)	10.26 μg/mL (WT) 110.15 μg/mL (Alpha) 59.74 μg/mL (Beta) 26.39 μg/mL (Delta) 2.86 μg/mL (BA.1) 4.92 μg/mL (BA.1.1) 0.73 μg/mL (BA.2) 1.54 μg/mL (BA.4) 1.91 μg/mL (BA.5) 3.13 μg/mL (BF.7) 0.4 μg/mL (XBB)	Alpha Beta Delta BA.1 BA.1.1 BA.2 BA.4 BA.5 BF.7 XBB	Protects Ad5-hACE2-transduced mice from SARS-CoV-2 Omicron XBB (pseudovirus) infection	145
S2A3	Phage display	SARS-CoV-2 (WT) S (Llama)	S2	0.56 nmol/L (WT) 2.18 nmol/L (Alpha) 0.85 nmol/L (Beta) a SPR	ND	Fc format: 12.2 nmol/L (WT) 31 nmol/L (Alpha) 54 nmol/L (Beta) 5.36 nmol/L (Omicron)	Alpha Beta Omicron	Fc format protects hamsters from SARS-CoV-2 WT infection	144
79C11	Phage display	SARS-CoV-2 S proteins of Omicron BA.2, BA.4 and BF.7 (Sharks)	S2	19–46 nmol/L (WT, Delta, BA.1, BA.2, BA.4, BF.7, BQ.1.1, XBB.1.5, XBB.1.16.1, EG.5, BA.2.86 and JN.1) a SPR	0.794 μg/mL (WT) 0.566 μg/mL (Delta) 0.610 μg/mL (BA.2) 0.640 μg/mL (BA.4) 0.575 μg/mL (BQ.1.1) 0.794 μg/mL (XBB.1) 0.719 μg/mL (JN.1) 0.549 μg/mL (KP.2)	ND	Delta BA.2 BA.4 BQ.1.1 XBB.1 JN.1 KP.2	Fc format protects K18-hACE2 transgenic mice from SARS-CoV-2 Omicron XBB.1 infection	146
S2A9	Phage display	Naïve library (Sharks)	S2	590 nmol/L (WT) a SPR	4134 nmol/L (WT) 1740 nmol/L (Alpha) 2144 nmol/L (Beta) 1711 nmol/L (Gamma) 1788 nmol/L (Delta) 2143 nmol/L (BA.1) 2248 nmol/L (BA.1.1)	3601 nmol/L (WT) 2136 nmol/L (Alpha) 704.3 nmol/L (Beta) 191 nmol/L (Gamma) 213.7 nmol/L (Delta) 663.3 nmol/L (BA.1) 1560 nmol/L (BA.1.1)	Alpha Beta Gamma Delta BA.1 BA.1.1	ND	147
**Nanobodies targeting non-S proteins of SARS-CoV-2 variants**									
2NSP23		SARS-CoV-2 NSP9 protein with C14S, C23S and C73S mutations (Llama)	NSP9	ND	LNP-mRNA-2NSP23: 1.8 ng/μL (for inhibiting SARS-CoV-2 WT (Hu-1) replication) 0.4 ng/μL (for inhibiting Alpha, Delta, Mu and Omicron variant replication)		Alpha Delta Mu Omicron	ND	149

a ACE2, angiotensin-converting enzyme 2; Ad5-hACE2 mice, adenovirus 5-transduced human ACE2 mice; BLI, Bio-Layer Interferometry; EC_50_, half-maximal effective concentration; hACE2-Tg mice, human ACE2-transgenic mice; IC_50_, half-maximal inhibitory concentration; LNP-mRNA-2NSP23: encapsulated 2NSP23 into lipid nanoparticles as mRNA; ND, not determined; N/S, not listed here; NSP9, non-structural protein 9; NTD, N-terminal domain; RBD, receptor-binding domain; S, spike; SARS-CoV-2, severe acute respiratory syndrome coronavirus-2; SPR, Surface Plasmon Resonance; VOCs, variants of concern stains; WT, wild-type.

**Figure 5:**
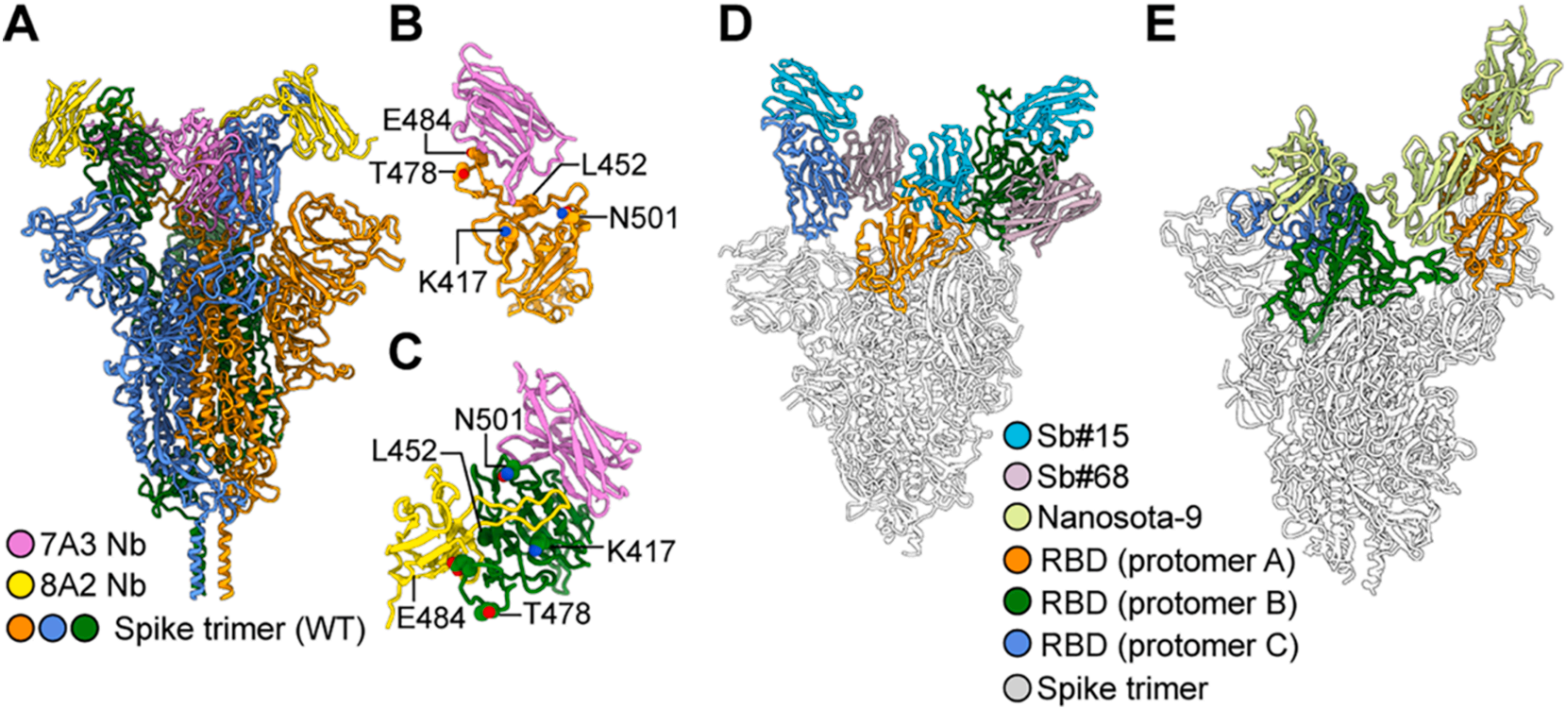
Cryo-EM structures of nanobodies targeting SARS-CoV-2 spike (S) or receptor-binding domain (RBD). (A) Cryo-EM structure of SARS-CoV-2 S in complex with nanobodies 7A3 and 8A2 (PDB 7TPR). (B) Closed-up view of the interactions between nanobody 7A3 and RBD from protomer A S subunit. Residues in RBD that are commonly mutated in variants are shown as spheres and labeled. (C) Closed-up view of the interactions among nanobodies 7A3, 8A2, and RBD from protomer B S subunit. Residues in RBD that are commonly mutated in variants are shown as spheres and labeled. (D) Cryo-EM structure of SARS-CoV-2 S in complex with nanobodies Sb#15 and Sb#68 (PDB 7P78). (E) Cryo-EM structure of SARS-CoV-2 Omicron variant S in complex with nanobody Nanosota-9 (PDB 9CO6).

SaRBD-1, a nanobody derived from a phage display library generated from SARS-CoV-2 RBD-immunized alpaca, was effective in neutralizing Alpha, Beta, Gamma, and Delta VOCs, with the binding affinity in the nanomolar or picomolar range^124^. It was also effective against the SARS-CoV-2 original strain. It binds to the RBD of SARS-CoV-2, thus interfering with the interaction between the RBD and the ACE2 receptor. 7A3 and 8A2 nanobodies with high affinity for the RBD that target non-overlapping epitopes were identified from a dromedary camel VHH phage display library. Cryo-EM structural analysis revealed that 7A3 targets a buried region extending from the S1 subunit to the apex of the S2 subunit, while 8A2, which targets the RBD *via* a long CDR3 loop, directly blocks RBD binding to ACE2 (Fig. 5A–C)^125^. Combinatorial treatment with 7A3 and 8A2 was more effective than treatment with either one alone, with half-maximal inhibitory concentration (IC_50_) values of 5, 1.5, and 0.13 nmol/L against Alpha, Beta, and Gamma VOCs, respectively. Of note, K18-hACE2-transgenic mice were developed as an ideal small animal model to display severe SARS-CoV-2 infection features, being widely used for the evaluation of the efficacy of COVID-19 therapies, among other applications^126^, ^127^, ^128^, ^129^. Indeed, it is shown that 7A3 protected K18-hACE2 transgenic mice from a lethal challenge with Beta or Delta VOC^125^. Additional nanobodies, H5, G7, and 2F4, were identified by immunizing a llama with the original SARS-CoV-2 RBD protein^130^. H5 neutralized both the pseudotyped original strain and Delta VOC but was ineffective against Omicron variants. 2F4 neutralized all pseudotyped pre-Omicron variants with similar efficacy but failed to show neutralizing activity against Omicron lineage variants. G7 was more than 5-fold less effective at neutralizing the pseudotyped Delta VOC than the original strain, and did not neutralize other variants^130^. ShAb01 and ShAb02 nanobodies were isolated by panning a phage display library derived from nurse sharks immunized with the SARS-CoV-2 RBD, RBD-ferritin, or S-ferritin nanoparticle protein. These nanobodies recognized distinct antigenic regions of the RBD and bound to the RBDs of Alpha, Beta, Gamma, and Delta VOCs with affinities in the low nanomolar range^131^. While ShAb01 neutralized Alpha, Beta, and Delta VOCs, as well as the original SARS-CoV-2 strain and SARS-CoV, with IC_50_ values of 188–873 ng/mL, ShAb02 neutralized Alpha, Beta, and Delta VOCs, as well as the SARS-CoV-2 original strain, with IC_50_ values of 15–52 ng/mL, but showed reduced neutralizing activity against SARS-CoV^131^.

Sb#15 and Sb#68 nanobodies were obtained by screening a synthetic library for nanobodies that bind to the RBD of the S protein of SARS-CoV-2 and block its interaction with S-ACE2. The nanobodies showed neutralizing activity against pseudotyped Alpha, Beta, and Delta VOCs with IC_50_ values of 84–299 nmol/L (for Sb#68)^132^. Cryo-EM structural analysis revealed that Sb#15 and Sb#68 are bound to two discrete epitopes in the RBD (Fig. 5D). Sb#15 bound to residues 444–448 and 491–507, whereas Sb#68 bound to residues 369–385 and R408 in the RBD^132^. RBD-1-2G, identified by screening synthetic humanized nanobody libraries, targets the RBD of the S protein and neutralizes Alpha VOC and the original strain of SARS-CoV-2, with IC_50_ values of 746.5 and 511.6 nmol/L, respectively^133^. Cryo-EM analysis indicated that it binds to the ‘up’ RBD position, as well as to the RBD in its ‘partial down’ conformation^133^.

In addition to nanobodies generated from immunized alpaca, camel, llama, and sharks, as well as from the synthetic libraries described above, recent advancements have made it possible to generate nanobodies from mice genetically engineered to produce camelid antibodies, termed nanomice^134^. Nb12 and Nb30 nanobodies, which were generated from immunized nanomice, target different regions of the RBD of the SARS-CoV-2 S protein. They show neutralizing activity against the pseudotyped and authentic SARS-CoV-2 original strain, as well as against Alpha, Beta, and Gamma VOCs, with bivalent Nb30 showing improved neutralizing activity against authentic Alpha and Beta VOCs (IC_50_ 538 and 2755 pmol/L, respectively)^134^.

### 6.2 Nanobodies targeting the RBD of SARS-CoV-2 Omicron variant and its subvariants

A number of RBD-targeting nanobodies have been identified with strong binding affinity and/or broadly neutralizing activity against multiple SARS-CoV-2 Omicron subvariants (Table 1). Nanosota-9, which was isolated from an immunized alpaca phage display library, showed strong binding to Omicron RBDs, with *K*_D_ values of 0.078, 0.06, and 29.4 nmol/L for the RBDs of Omicron BA.5, XBB.1.5, and JN.1, respectively, as measured by Surface Plasmon Resonance (SPR)^135^. It broadly neutralized these Omicron subvariants in both their pseudotyped and authentic versions. Cryo-EM structural analysis indicated that Nanosota-9 neutralized the Omicron variant by crosslinking two RBDs of the S protein, thereby preventing the RBDs from interacting with the ACE2 receptor (Fig. 5E). The binding epitopes of Nanosota-9 on the S protein are relatively conserved among different Omicron subvariants, contributing to the broad-spectrum efficacy of this nanobody against these variants^135^. Other alpaca-derived nanobodies, such as A14, bind to Omicron BA.1 RBD with a *K*_D_ value of 0.065 nmol/L (estimated by SPR) and broadly neutralize Omicron BA.1, BA.4/5, BQ.1.1, and XBB.1, as well as the SARS-CoV-2 original strain, with IC_50_ values of 5.49–20.69 nmol/L^136^. H6 and B5-5 nanobodies targeting the Omicron RBD were isolated from immunized llama phage display libraries. They neutralize Omicron XBB.1.5 and BA.5, with IC_50_ values of 1.32 and 0.219 nmol/L, respectively, in the H6 trimer version, and IC_50_ values of 262 and 352 nmol/L, respectively, in the B5-5 trimer version^137^. While H6 binds to conserved residues at the ACE2-RBD interface to block RBD binding to the ACE2, B5-5 binds to conserved residues in RBD that do not overlap with the ACE2 binding region. Intranasal administration of trimeric versions of both nanobodies prophylactically and/or therapeutically protected hamsters from Omicron BA.5 infection^137^. Nb1 and Nb2 nanobodies showed broad-spectrum anti-Omicron neutralizing activity, neutralizing 10 Omicron subvariants, including BA.1, BA.2, BA.3, BA.5, BA.2.75, BF.7, BQ.1, EG.5.1, XBB.1.5, and JN.1, in addition to the SARS-CoV-2 original strain^138^. Molecular dynamics simulations showed that Nb1 and Nb2 formed stable structures with the S protein through hydrogen bonds and hydrophobic interactions involving the CDR1 and CDR2 regions. The key RBD residues that are crucial for nanobody binding are S375, K378, D405, and G446^138^.

**Figure 6:**
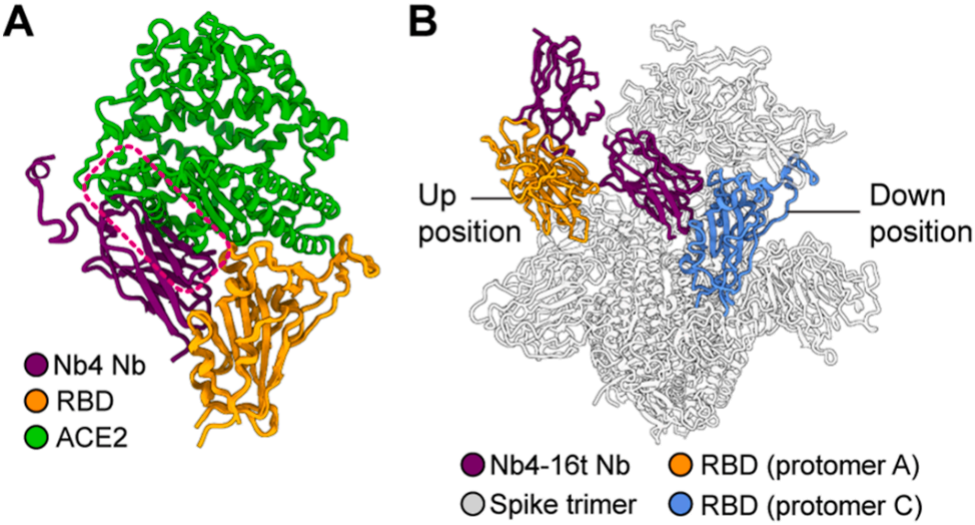
Recognition of SARS-CoV-2 Omicron spike (S) proteins by nanobody Nb4. (A) Superimposition of the crystal structures of SARS-CoV-2 Omicron BA.1 subvariant receptor-binding domain (RBD) in complex with either ACE2 (PDB 7WPB) or nanobody Nb4 (PDB 8K3K). The potential region of steric clashes between ACE2 and nanobody Nb4 is highlighted by a magenta-dashed box. (B) Cryo-EM structure of SARS-CoV-2 Omicron BA.5 subvariant S trimer in complex with nanobody Nb4-16t (PDB 8K47). Only one nanobody domain of the trivalent Nb4-16t is visible in the cryo-EM structure. RBDs in the “up” and “down” positions, respectively, are indicated.

Other RBD-targeting nanobodies were reported to bind with high affinity to and/or to broadly neutralize multiple SARS-CoV-2 Omicron subvariants and early VOCs (Table 1). Several Omicron BA.1 RBD-targeting nanobodies, which were identified by screening a naive alpaca VHH library, exhibit high binding affinity for the RBD of Omicron BA.1 and BA.2, as well as early VOCs (Alpha, Beta, and Delta) and the original strain^139^. The Re32D03 nanobody, isolated from an immunized alpaca phage display library, binds to Omicron BA.1, BA.2.75, BA.5, and BQ.1, in addition to Delta VOC, and neutralizes pseudotyped Alpha, Delta, and Omicron BA.2.75 VOCs^140^. The crystal structure of the Re32D03 nanobody in complex with the RBD of SARS-CoV-2 Omicron BA.2.75 variant showed that it clearly binds to residues within the RBD of this variant^140^. Nanobody Nb4, derived from a synthetic library, or its trimeric form, neutralizes SARS-CoV-2 Delta VOC (IC_50_ 22 μg/mL) and several Omicron subvariants, such as BA.1.1 and BA.2.3, with IC_50_ values ranging from 0.4 to 2 μg/mL. Crystal structural analysis revealed that Nb4 competitively binds to an epitope on the RBD that partially overlaps with the ACE2 binding site (Fig. 6A), indicating that this is probably the mechanism responsible for its neutralizing activity^141^. The cryo-EM structure of the Nb4 trimer (Nb4-16t) in complex with Omicron BA.5 S protein indicated that Nb4 binds RBD in both its up and down positions (Fig. 6B)^141^. Intranasal administration of the Nb4 trimer protects wild-type mice from Omicron (BA.1.1, BA.2.3, and BA.5) infection prophylactically and therapeutically, with significantly reduced viral loads in the lungs^141^.

Some RBD-targeting nanobodies show broad neutralizing activity against not only SARS-CoV-2 Omicron variants, but also early VOCs and/or other CoVs (Table 1). Tnb04-1, isolated from an immunized alpaca yeast display library, shows broad neutralizing activity against Omicron subvariants, such as EG.5.1, HK.3, HV.1, JD.1.1, BA.2.86, and JN.1, with an average IC_50_ of 0.017 μg/mL, in addition to SARS-CoV (IC_50_ 0.005 μg/mL)^142^. It binds to a conserved hydrophobic pocket in the RBD of the S protein that lies outside the ACE2-binding site, preventing infection by interfering with the development of a proteinase K-resistant core essential for viral-cell fusion. Intranasal administration of this nanobody effectively protected the respiratory tract of Syrian hamsters from infection with the Omicron XBB.1.5 subvariant^142^ and prevented them from transmitting this variant^142^.

The sequential immunization of animals, such as llamas, has also been used to generate broadly RBD-targeting nanobodies (Table 1). For example, sequential immunization of a llama with the RBD of the original strain, followed by immunization in the order with Beta and Delta RBD variants of SARS-CoV-2, SARS-CoV RBD, and finally with SARS-CoV-2 Omicron BA.5 RBD, generated nanobodies with broad neutralizing activity against several variants, including Omicron subvariants^130^. Using this approach, 9A7, 9A21, 9A57, and 9A58 nanobodies were isolated and shown to neutralize pseudotyped Omicron subvariants (BA.5, BQ.1, XBB.1), in addition to the original strain, with IC_50_ values of 0.5–29.4 nmol/L. The Fc versions of these nanobodies showed improved neutralizing activity against other Omicron subvariants, such as BA.1, BA.2, XBB.1.5, and XBB.1.16^130^.

### 6.3 Nanobodies targeting the non-RBD spike protein of SARS-CoV-2 variants

A set of nanobodies that bind to non-RBD regions in S protein, including the NTD and/or S2 subunit, of SARS-CoV-2 S proteins has been identified, some of which show broad neutralizing activity. These nanobodies most likely inhibit S protein function by obstructing the conformational changes required for the correct functioning of the S protein or by blocking the S-mediated membrane fusion process (Table 1).

**Figure 7:**
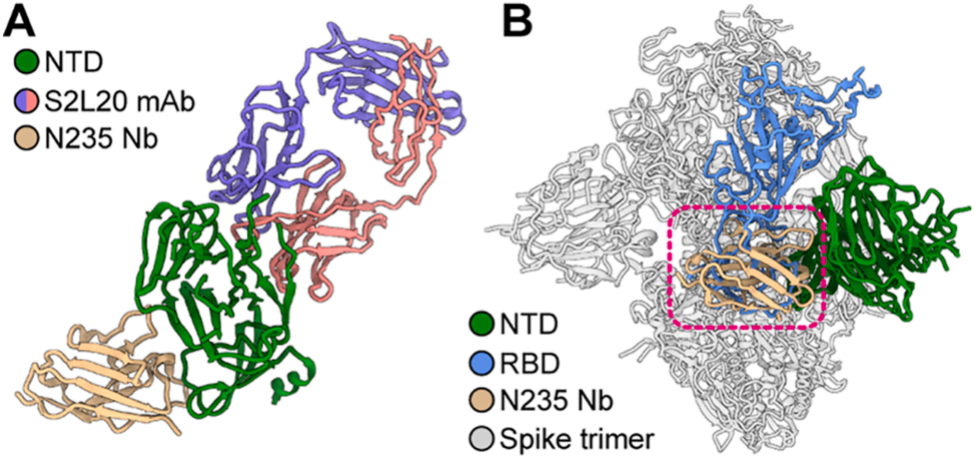
SARS-CoV-2 spike (S) N-terminal domain (NTD)-recognizing nanobody. (A) Cryo-EM structure of SARS-CoV-2 Omicron BA.1 subvariant S-NTD in complex with nanobody N235 (PDB 8JVA). (B) Superimposition of the of SARS-CoV-2 Omicron BA.1 subvariant NTD–N235 complex structure to the structure of SARS-CoV-2 Omicron variant S trimer (PDB 7WK2). The region of steric clashes between nanobody N235 and S-receptor-binding domain (RBD) is highlighted by a magenta-dashed box.

Nanobodies targeting the NTD of SARS-CoV-2 variants have been reported. For example, an alpaca-derived nanobody, N235, binds to various SARS-CoV-2 variants, including multiple Omicron subvariants (*i*.*e*., BA.1, BA.2, BA.2.75, BA.3, BA.5, XBB, XBB.1.5, XBB.1.16, EG.5.1, and BA.2.86), as well as early VOCs (*i*.*e*., Alpha, Beta, Gamma, and Delta variants), and broadly neutralizes infection with pseudotyped versions of many of these variants^143^. Cryo-EM structural and mechanistic analyses have shown that N235 binds to a conserved cryptic epitope in the NTD that interferes with the functioning of the RBD of the S protein (Fig. 7A and B), leading to the shedding of the S1 subunit from the trimeric S complex and disruption of the ability of the virus to infect cells^143^. A llama-derived nanobody SR01 targets the NTD and binds to early SARS-CoV-2 variants (Alpha and Beta) and SARS-CoV, with its Fc version showing neutralization potency from 3.77 to 70.3 nmol/L against live SARS-CoV-2 VOCs, including Alpha, Beta, and Omicron^144^. The Fc-fused SR01 also prophylactically protects hamsters from infection with the SARS-CoV-2 original authentic strain^144^.

Nanobodies targeting the S2 region of SARS-CoV-2 variants have also been developed, some of which exhibit broad neutralizing activity. For example, an alpaca-derived nanobody S102, which targets the S2 subunit of sarbecoviruses, including SARS-CoV-2, broadly neutralizes various SARS-CoV-2 variants, such as the immune-evading Omicron subvariants BQ.1.1 and XBB, as well as other sarbecoviruses, such as SARS-CoV and RaTG13^145^. The IC_50_ value of this nanobody against the pseudotyped SARS-CoV-2 original strain and various variants ranges from 0.37 to 5.68 μg/mL for the SARS-CoV-2 original strain and previous VOCs (*i*.*e*., Alpha, Beta, Gamma, and Delta), and from 0.52 to 0.95 μg/mL for Omicron subvariants, such as BA.1, BA.2, BA.4/5, BQ.1, BQ.1.1, BF.7, and XBB. It also neutralizes early and Omicron variants of live SARS-CoV-2 with various IC_50_ values. This nanobody functions by inhibiting the cell membrane fusion process through stabilizing the stem helix region near the HR2 region, preventing the structural changes necessary for the formation of the six-helix bundle that is crucial for virus entry into host cells^145^. Another alpaca-derived nanobody that targets the S2 subunit, S2A3, binds to early VOCs of SARS-CoV-2, including Alpha and Beta, as well as SARS-CoV, and its Fc version neutralizes live SARS-CoV-2 VOCs, such as Alpha, Beta, and Omicron, with IC_50_ values of 5.36–54 nmol/L^144^. S2A3-Fc prophylactically protects hamsters from SARS-CoV-2 challenge (original authentic strain) and reduces lung viral titers^144^. A shark-derived nanobody, 79C11, which binds to a conserved region in the HR1 of the S2 subunit of sarbecovirus S proteins, neutralizes the pseudotyped SARS-CoV-2 original strain, Delta VOC, and multiple Omicron subvariants, such as BA.2, BA.4, BQ.1.1, XBB.1, JN.1, and KP.2, with IC_50_ values of 0.549–0.794 μg/mL, as well as SARS-CoV and pangolin CoV, with IC_50_ values of 0.57 and 0.649 μg/mL, respectively^146^. Intranasal administration of this nanobody or its Fc version prophylactically protected K18-hACE2 transgenic mice from infection with the Omicron XBB.1 subvariant^146^. Another shark-derived nanobody, S2A9, targets the S2 subunit of SARS-CoV-2 or cross-reacts with S2 of other beta-coronaviruses (*i*.*e*., SARS-CoV or MERS-CoV)^147^. It neutralizes pseudotyped SARS-CoV-2 Alpha, Beta, Gamma, Delta, and Omicron VOCs (BA.1, BA.2, BA.4/5), with IC_50_ values of 1.71–4.36 μmol/L, as well as live SARS-CoV-2 Alpha, Beta, Gamma, Delta, or Omicron BA.1 and BA.1.1 variants, with IC_50_ values of 0.191–2.14 μmol/L^147^. Although an Fc version of this nanobody (S2A9-Fc) increases neutralizing activity against some pseudotyped SARS-CoV-2 variants or SARS-CoV *in vitro*, its efficacy *in vivo* remains unknown^147^. Other S2-targeting nanobodies, such as S2-1, S2-10, S2-40, and S2-62, do not show potent neutralization against SARS-CoV-2 infection *in vitro*^148^.

### 6.4 Nanobodies targeting non-S proteins of SARS-CoV-2 variants

Only a few nanobodies targeting non-S proteins of SARS-CoV-2 variants have been reported (Table 1). The 2NSP23 nanobody, which targets the NSP9, blocks SARS-CoV-2 replication *in vitro*^149^. Its lipid nanoparticle-encapsulated mRNA (*i*.*e*., LNP-mRNA-2NSP23) suppresses viral replication of several SARS-CoV-2 variants, including Alpha, Delta, and Omicron^149^. Other nanobodies, such as those targeting the conserved N protein, do not show neutralizing activity against viral infection, but are useful for diagnostic purposes^150^.

## 7 Improving potency and/or half-life of SARS-CoV-2 variant-targeting nanobodies

Mutations in the S protein, particularly the RBD, can lead to resistance against neutralizing nanobodies/antibodies by altering key structural and functional features required for binding^151^. Multivalent nanobody formats introduced below represent a powerful strategy to overcome RBD mutation-mediated immune escape. By leveraging avidity through simultaneous engagement of multiple RBDs or distinct epitopes, these constructs maintain neutralizing activity against variants harboring key escape substitutions. Multivalent binding increases the genetic barrier to resistance and compensates for affinity losses caused by individual mutations, thereby enabling sustained neutralization of highly divergent SARS-CoV-2 sublineages.

Multivalent nanobodies, such as bispecific, dimeric, or trimeric formats, have been developed to improve the efficiency of SARS-CoV-2 variant-targeting nanobodies, some of which are resistant to S/RBD mutations. For example, the RBD-targeting biparatopic Nb1-Nb2 nanobody has high affinity and neutralizing activity against several live viruses of early SARS-CoV-2 VOCs, including Alpha, Beta, Gamma, and Delta, with IC_50_ values ranging from 0.732 to 13.01 nmol/L^152^. This biparatopic nanobody exhibits remarkable resistance to RBD mutation-mediated escape, retaining neutralization against major substitutions including K417N/T, E484A/K, L452R, T478K, N501Y, Q493R, and Q498R^152^. The biparatopic nanobodies NM1267 and NM1268 target different epitopes inside the RBD–ACE2 interface^153^. Both nanobodies show strong RBD binding affinity, efficiently binding to Alpha, Beta, Gamma, and Delta VOCs. NM1267 neutralizes Beta and Delta VOCs, with IC_50_ values of 0.78 and 52.55 nmol/L, respectively, and NM1268 also neutralizes Beta and Delta VOCs, with IC_50_ values of 6.06 and 0.67 nmol/L, respectively. Intranasal treatment with NM1267 and NM1268 prophylactically inhibits the infection of K18-hACE2 transgenic mice with early Beta and Delta VOCs, as well as SARS-CoV-2 B.1, and attenuates virus shedding, disease progression, and mortality^153^. While the P2C5 monomer neutralizes Alpha, Beta, Gamma, and Omicron VOCs but not Delta VOC, monomeric forms of P5F8 and P2G1 are active against all other VOCs tested, except Omicron VOC^154^. Notably, their dimeric versions show a 200-fold increase in neutralizing activity. A heterodimeric nanobody, P2C5-P5F8, efficiently neutralizes all SARS-CoV-2 VOCs tested, including Alpha, Beta, Gamma, Delta, and Omicron variants, with IC_50_ values ranging from 2.85 to 709 nmol/L^154^. Bivalent and trivalent versions of RBD-1-2G nanobodies exhibit increased viral neutralization potency, with IC_50_ values of 88 and 4.1 nmol/L, respectively^133^. While the S2-targeting monomeric nanobodies S2-7 and S2-10 show low or no neutralizing activity against pseudotyped SARS-CoV-2, their dimeric and trimeric versions have improved neutralization potency^148^.

Multivalent nanobodies, including tetravalent and hexavalent, have also been constructed to overcome escape mutations and increase neutralization potency against SARS-CoV-2 variants. While bivalent VHH-72 has modest neutralization activity (IC_50_ 3.3 nmol/L) against SARS-CoV-2, its tetravalent form increases neutralization potency with an IC_50_ value of 0.34 nmol/L^155^. Hexavalent VHH-72 exhibits even better neutralization potency with an IC_50_ value of 0.035 nmol/L, potently neutralizing both Alpha (IC_50_ 0.31 nmol/L) and Beta (IC_50_ 0.072 nmol/L) VOCs^155^. The multivalent VHH-72 nanobodies retain activity against major escape substitutions, including K417N/T, E484K, L452R, T478K, and N501Y. These multivalent nanobodies have favorable drug-like biophysical properties, such as high solubility, strong stability, and low-level non-specific binding^155^.

To further improve the potency and/or half-life of SARS-CoV-2 variant-targeting nanobodies, nanobodies are often fused to an Fc tag to constitute a different multivalent format of nanobodies. Indeed, a variety of Fc-fused, SARS-CoV-2-targeting nanobodies have been developed that show improved binding affinity, broad neutralizing activity, and/or increased half-life. For example, saRBD-1 is engineered to generate an Fc-conjugated nanobody construct that presents improved binding and neutralization of early SARS-CoV-2 variants, including Alpha, Beta, Gamma, and Delta VOCs^124^. An engineered nano-IgM construct, MN235, generated by fusing the N235 nanobody to the Fc region of human IgM, exhibits higher neutralizing potency than monomeric N235 alone, effectively preventing infection by pseudotyped and authentic viruses^143^. Intranasal administration of MN235 prophylactically prevents infection caused by pseudotyped Omicron subvariants XBB and BA.1 in adenovirus 5-expressing human ACE2 mice^143^. A human IgG1 Fc-conjugated construct, Nb1-Nb2-Fc, exhibits improved neutralization potency against pseudotyped and/or live Alpha, Beta, Gamma, Delta, and Omicron VOCs^152^. Notably, addition of an Fc tag to Nb1 and Nb2 nanobodies efficiently increases their broad-spectrum neutralizing activity against multiple authentic SARS-CoV-2 variants, in addition to the original strain, with IC_50_ values of 23.57, 121.1, 179.5, and 169 ng/mL against the original strain and Omicron BA.5, BQ.1, and EG.5.1 subvariants, respectively, for Nb1-Fc, and IC_50_ values of 520.7, 1269, 3332, and 1165 ng/mL against the original strain and Omicron BA.5, BQ.1, and EG.5.1 subvariants, respectively, for Nb2-Fc^138^. Nanosota-9-Fc binds strongly to the S ectodomains of various Omicron subvariants, including BA.5, XBB.1.5, and JN.1, and neutralizes Omicron BA.5, XBB.1.5, and JN.1 live viruses with IC_50_ values of 0.010, 0.098, and 0.044 μg/mL, respectively^135^. It also effectively protects wild-type mice from infection with these Omicron variants^135^. The Fc-conjugated nanobodies, aRBD-2-5-Fc and aRBD-2-7-Fc, neutralize all pseudotyped and live SARS-CoV-2 tested, including the original strain, Alpha, Beta, Gamma, Delta, and Omicron BA.1, BA.1.1 and BA.2, with IC_50_ values of 0.0830, 0.0438, 0.0271, and 0.0293 nmol/L, respectively, against the original strain, Beta, Delta, and Omicron BA.1 for aRBD-2-5-Fc, and IC_50_ values of 0.0389, 0.1031 and 0.0534, and 0.1299 nmol/L, respectively, for aRBD-2-7-Fc^156^. aRBD-2-5-Fc prophylactically protects against infection with the original strain and mouse-adapted SARS-CoV-2 in mice, and confers protection against the Omicron BA.1 variant in hamsters prophylactically and therapeutically^156^. Pharmacokinetic studies indicate that aRBD-2-5-Fc has a long half-life of 165 h in mice and 72 h in hamsters^156^.

Other multivalent nanobody formats can be constructed by fusing SARS-CoV-2 variant-targeting nanobodies to human serum albumin (HSA) protein or HSA-targeting nanobodies, based on which to improve the binding affinities of nanobodies, resulting in broader neutralizing activity and/or a longer half-life. For instance, the trimeric nanobody Nb_15_-Nb_H_-Nb_15_, consisting of two molecules of the SARS-CoV-2 RBD-targeting Nb15 nanobody and one HSA-specific nanobody, has high neutralization potency against the pseudotyped original strain, Alpha, and Delta VOCs of SARS-CoV-2, with IC_50_ values ranging from 0.26 to 5.16 ng/mL^157^. Intranasal administration of this nanobody results in the nanobody reaching the lungs of mice and persisting for 7 days. This trimeric nanobody protects hACE2 transgenic mice against SARS-CoV-2 infection prophylactically and therapeutically^157^.

## 8 Conclusions and future perspectives

Nanobody-based therapeutics have emerged as a promising class of biologics with distinct structural and functional advantages over conventional antibodies and other therapeutic agents. The small size, high stability, cryptic-epitope accessibility, and low-cost production of nanobodies expand their therapeutic landscape for infectious diseases, oncology, inflammation, neurological disorders, and much more. Although nanobody technology has matured substantially over the last decade, highlighted by the approval of caplacizumab as the first nanobody-derived drug to treat acquired thrombotic thrombocytopenic purpura, several considerations remain critical for the broad clinical implementation of nanobodies^158^^,^^159^. The short systemic half-life of nanobodies stemming from their small size and fast renal clearance still complicates dosing regimens, although albumin fusion, Fc fusion, PEGylation, or multimerization strategies offer significant solutions^160^. In addition, immunogenicity of nanobodies could be a potential concern to some extent, particularly for camelid-derived sequences; thus, extensive humanization and computational redesign of nanobodies are essential to minimize anti-drug antibody responses^161^. Delivery may also present some obstacles: while nanobodies are highly stable, achieving efficient tissue penetration in certain compartments, such as the central nervous system, requires improved carrier systems^162^. Furthermore, the regulatory issue related to next-generation constructs through incorporating nanobody recognition domains, including CAR-T designs and nanobody-drug conjugates, is still evolving, which may extend the timelines for approval^104^.

As described above, nanobodies are promising candidates for the prevention and treatment of SARS-CoV-2. Several nanobodies with high neutralization potency and/or broad neutralization spectrums have been developed against SARS-CoV-2 variants, including recent Omicron subvariants, and/or other sarbecoviruses (such as SARS-CoV). This review article brings together information on the major proteins of SARS-CoV-2 and their functions, the viral life cycle, and S protein-mediated viral entry and fusion, and describes differences among SARS-CoV-2 variants. It comprehensively summarizes the properties of SARS-CoV-2-targeting nanobodies, including those that target the RBD of early variants or the Omicron variant and its subvariants, the non-RBD S protein of such variants, and the non-S proteins of SARS-CoV-2 variants. Finally, this review discusses the various approaches used to increase the potency and/or half-life of SARS-CoV-2 variant-targeting nanobodies.

Because of the differences in detection assays, calculation methods, nanobody doses/routes, virus strains/titers, and/or animal models used to characterize nanobodies, some nanobodies may turn out to have lower or higher potency than others. Nevertheless, most reported nanobodies target the S protein of SARS-CoV-2, particularly the RBD. Compared with other regions (*i*.*e*., NTD and S2) of the S protein, the RBD is subject to far more mutations, potentially leading to additional variants that might escape currently available nanobodies. Therefore, it is critical to develop nanobodies with high potency and broad neutralization spectrums that target conserved residues in the RBD and highly conserved regions in the NTD and S2 of the SARS-CoV-2 S protein to prevent and treat infections with current and future SARS-CoV-2 variants, in addition to other sarbecoviruses with pandemic potential. In this respect, it is encouraging that dimeric, trimeric, and/or multivalent nanobodies show improved neutralization potency against SARS-CoV-2 variants and/or other sarbecoviruses. One approach that could be used to further improve the neutralization spectrum of these nanobodies would be to alter them so that they target two or multiple conserved epitopes in the RBD, NTD, and/S2 of SARS-CoV-2 variants. In the near future, it is anticipated that more SARS-CoV-2-targeting nanobodies with higher efficacy and broad neutralizing activity will enter human clinical trials, hopefully leading to the development of novel therapeutics for the management of COVID-19.

## Author contributions

Abhijeet Roy and Yang Yang: Figure preparation. Abhijeet Roy and Lanying Du: Writing – original draft. Lanying Du: Writing – review and editing. Yang Yang and Lanying Du: Funding acquisition. Abhijeet Roy, Yang Yang, and Lanying Du: Validation.

## Declaration of generative AI in scientific writing

No artificial intelligence (AI) and AI-assisted technologies are used in this manuscript.

## Conflicts of interest

The authors declare no conflicts of interest.

## References

[1] Stadler K., Masignani V., Eickmann M., Becker S., Abrignani S., Klenk H.D. (2003). SARS-beginning to understand a new virus. Nat Rev Microbiol.

[2] Zhong N.S., Zheng B.J., Li Y.M., Poon L.L.M., Xie Z.H., Chan K.H. (2003). Epidemiology and cause of severe acute respiratory syndrome (SARS) in Guangdong, people’s Republic of China, in February, 2003. Lancet.

[3] Zaki A.M., van Boheemen S., Bestebroer T.M., Osterhaus A.D., Fouchier R.A. (2012). Isolation of a novel coronavirus from a man with pneumonia in Saudi Arabia. N Engl J Med.

[4] European Center for Disease Prevention and Control MERS-CoV worldwide overview. https://www.ecdc.europa.eu/en/middle-east-respiratory-syndrome-coronavirus-mers-cov-situation-update.

[5] Cucinotta D., Vanelli M. (2020). WHO declares COVID-19 a pandemic. Acta Biomed.

[6] Zhu N., Zhang D., Wang W., Li X., Yang B., Song J. (2020). A novel coronavirus from patients with pneumonia in China, 2019. N Engl J Med.

[7] World Health Organization. WHO COVID-19 dashboard [cited April 26, 2026]. Available from: https://data.who.int/dashboards/covid19/cases.

[8] Carabelli A.M., Peacock T.P., Thorne L.G., Harvey W.T., Hughes J., Peacock S.J. (2023). SARS-CoV-2 variant biology: immune escape, transmission and fitness. Nat Rev Microbiol.

[9] You Y., Yang X., Hung D., Yang Q., Wu T., Deng M. (2024). Asymptomatic COVID-19 infection: diagnosis, transmission, population characteristics. BMJ Support Palliat Care.

[10] Amicone M., Borges V., Alves M.J., Isidro J., Zé-Zé L., Duarte S. (2022). Mutation rate of SARS-CoV-2 and emergence of mutators during experimental evolution. Evol Med Public Health.

[11] Zhou L., Ayeh S.K., Chidambaram V., Karakousis P.C. (2021). Modes of transmission of SARS-CoV-2 and evidence for preventive behavioral interventions. BMC Infect Dis.

[12] Struyf T., Deeks J.J., Dinnes J., Takwoingi Y., Davenport C., Leeflang M.M. (2022). Signs and symptoms to determine if a patient presenting in primary care or hospital outpatient settings has COVID-19. Cochrane Database Syst Rev.

[13] Thaweethai T., Donohue S.E., Martin J.N., Hornig M., Mosier J.M., Shinnick D.J. (2025). Long COVID trajectories in the prospectively followed RECOVER-adult US cohort. Nat Commun.

[14] Ballering A.V., van Zon S.K.R., Olde Hartman T.C., Rosmalen J.G.M. (2022). Persistence of somatic symptoms after COVID-19 in the Netherlands: an observational cohort study. Lancet.

[15] Aiyegbusi O.L., Hughes S.E., Turner G., Rivera S.C., McMullan C., Chandan J.S. (2021). Symptoms, complications and management of long COVID: a review. J R Soc Med.

[16] Mahdy M.A.A., Younis W., Ewaida Z. (2020). An overview of SARS-CoV-2 and animal infection. Front Vet Sci.

[17] Peng M. (2020). Outbreak of COVID-19: an emerging global pandemic threat. Biomed Pharmacother.

[18] Luo C.H., Morris C.P., Sachithanandham J., Amadi A., Gaston D., Li M. (2021). Infection with the SARS-CoV-2 Delta variant is associated with higher infectious virus loads compared to the alpha variant in both unvaccinated and vaccinated individuals. medRxiv.

[19] Mohsin M., Mahmud S. (2022). Omicron SARS-CoV-2 variant of concern: a review on its transmissibility, immune evasion, reinfection, and severity. Medicine.

[20] Naqvi A.A.T., Fatima K., Mohammad T., Fatima U., Singh I.K., Singh A. (2020). Insights into SARS-CoV-2 genome, structure, evolution, pathogenesis and therapies: structural genomics approach. Biochim Biophys Acta Mol Basis Dis.

[21] Abdelrahman Z., Li M., Wang X. (2020). Comparative review of SARS-CoV-2, SARS-CoV, MERS-CoV, and influenza A respiratory viruses. Front Immunol.

[22] Pollett S., Conte M.A., Sanborn M., Jarman R.G., Lidl G.M., Modjarrad K. (2021). A comparative recombination analysis of human coronaviruses and implications for the SARS-CoV-2 pandemic. Sci Rep.

[23] Zhang Q., Guo H.L., Wang J., Zhang Y., Deng P.J., Li F.F. (2022). Structural genomic analysis of SARS-CoV-2 and other coronaviruses. Front Genet.

[24] Saxena S.K., Kumar S., Baxi P., Srivastava N., Puri B., Ratho R.K. (2020). Chasing COVID-19 through SARS-CoV-2 spike glycoprotein. Virusdisease.

[25] Bai C., Zhong Q., Gao G.F. (2022). Overview of SARS-CoV-2 genome-encoded proteins. Sci China Life Sci.

[26] Yang H., Rao Z. (2021). Structural biology of SARS-CoV-2 and implications for therapeutic development. Nat Rev Microbiol.

[27] Gordon D.E., Jang G.M., Bouhaddou M., Xu J., Obernier K., White K.M. (2020). A SARS-CoV-2 protein interaction map reveals targets for drug repurposing. Nature.

[28] Walls A.C., Park Y.J., Tortorici M.A., Wall A., McGuire A.T., Veesler D. (2020). Structure, function, and antigenicity of the SARS-CoV-2 spike glycoprotein. Cell.

[29] Wrapp D., Wang N., Corbett K.S., Goldsmith J.A., Hsieh C.L., Abiona O. (2020). Cryo-EM structure of the 2019-nCoV spike in the prefusion conformation. Science.

[30] Shang J., Ye G., Shi K., Wan Y., Luo C., Aihara H. (2020). Structural basis of receptor recognition by SARS-CoV-2. Nature.

[31] Lan J., Ge J., Yu J., Shan S., Zhou H., Fan S. (2020). Structure of the SARS-CoV-2 spike receptor-binding domain bound to the ACE2 receptor. Nature.

[32] Xia S., Liu M., Wang C., Xu W., Lan Q., Feng S. (2020). Inhibition of SARS-CoV-2 (previously 2019-nCoV) infection by a highly potent pan-coronavirus fusion inhibitor targeting its spike protein that harbors a high capacity to mediate membrane fusion. Cell Res.

[33] Huang Y., Yang C., Xu X.F., Xu W., Liu S.W (2020). Structural and functional properties of SARS-CoV-2 spike protein: potential antivirus drug development for COVID-19. Acta Pharmacol Sin.

[34] Cai Y., Zhang J., Xiao T., Peng H., Sterling S.M., Walsh R.M. (2020). Distinct conformational states of SARS-CoV-2 spike protein. Science.

[35] Gong Y., Qin S., Dai L., Tian Z. (2021). The glycosylation in SARS-CoV-2 and its receptor ACE2. Signal Transduct Targeted Ther.

[36] Yadav R., Chaudhary J.K., Jain N., Chaudhary P.K., Khanra S., Dhamija P. (2021). Role of structural and non-structural proteins and therapeutic targets of SARS-CoV-2 for COVID-19. Cells.

[37] Jahirul Islam M., Nawal Islam N., Siddik Alom M., Kabir M., Halim M.A. (2023). A review on structural, non-structural, and accessory proteins of SARS-CoV-2: highlighting drug target sites. Immunobiology.

[38] Mahtarin R., Islam S., Islam M.J., Ullah M.O., Ali M.A., Halim M.A. (2022). Structure and dynamics of membrane protein in SARS-CoV-2. J Biomol Struct Dyn.

[39] Khan F.I., Kang T., Ali H., Lai D. (2021). Remdesivir strongly binds to RNA-dependent RNA polymerase, membrane protein, and main protease of SARS-CoV-2: indication from molecular modeling and simulations. Front Pharmacol.

[40] Zhou S., Lv P., Li M., Chen Z., Xin H., Reilly S. (2023). SARS-CoV-2 E protein: pathogenesis and potential therapeutic development. Biomed Pharmacother Biomedecine Pharmacother.

[41] Mohammed M.E.A. (2021). The percentages of SARS-CoV-2 protein similarity and identity with SARS-CoV and BatCoV RaTG13 proteins can be used as indicators of virus origin. J Protein Proteonomics.

[42] Huang Y., Chen J., Chen S., Huang C., Li B., Li J. (2024). Molecular characterization of SARS-CoV-2 nucleocapsid protein. Front Cell Infect Microbiol.

[43] Zhou R., Zeng R., von Brunn A., Lei J. (2020). Structural characterization of the C-terminal domain of SARS-CoV-2 nucleocapsid protein. Mol Biomed.

[44] Yan R., Zhang Y., Li Y., Xia L., Guo Y., Zhou Q. (2020). Structural basis for the recognition of SARS-CoV-2 by full-length human ACE2. Science.

[45] Hoffmann M., Kleine-Weber H., Schroeder S., Krüger N., Herrler T., Erichsen S. (2020). SARS-CoV-2 cell entry depends on ACE2 and TMPRSS2 and is blocked by a clinically proven protease inhibitor. Cell.

[46] Hui K.P.Y., Ho J.C.W., Cheung M.C., Ng K.C., Ching R.H.H., Lai K.L. (2022). SARS-CoV-2 omicron variant replication in human bronchus and lung ex vivo. Nature.

[47] Nazir F., John Kombe Kombe A., Khalid Z., Bibi S., Zhang H., Wu S. (2024). SARS-CoV-2 replication and drug discovery. Mol Cell Probes.

[48] V’kovski P., Kratzel A., Steiner S., Stalder H., Thiel V. (2021). Coronavirus biology and replication: implications for SARS-CoV-2. Nat Rev Microbiol.

[49] Leung K., Shum M.H., Leung G.M., Lam T.T., Wu J.T. (2021). Early transmissibility assessment of the N501Y mutant strains of SARS-CoV-2 in the United Kingdom, October to November 2020. Euro Surveill.

[50] Zhang L., Mann M., Syed Z.A., Reynolds H.M., Tian E., Samara N.L. (2021). Furin cleavage of the SARS-CoV-2 spike is modulated by O-glycosylation. Proc Natl Acad Sci U S A.

[51] Maison D.P., Ching L.L., Shikuma C.M., Nerurkar V.R. (2021). Genetic characteristics and phylogeny of 969-bp S gene sequence of SARS-CoV-2 from Hawai‘i reveals the worldwide emerging P681H mutation. Hawaii J Health Soc Welf.

[52] Meng B., Kemp S.A., Papa G., Datir R., Ferreira I.A.T.M., Marelli S. (2021). Recurrent emergence of SARS-CoV-2 spike deletion H69/V70 and its role in the Alpha variant B.1.1.7. Cell Rep.

[53] Tegally H., Wilkinson E., Giovanetti M., Iranzadeh A., Fonseca V., Giandhari J. (2021). Detection of a SARS-CoV-2 variant of concern in South Africa. Nature.

[54] Salehi-Vaziri M., Fazlalipour M., Seyed Khorrami S.M., Azadmanesh K., Pouriayevali M.H., Jalali T. (2022). The ins and outs of SARS-CoV-2 variants of concern (VOCs). Arch Virol.

[55] Radvak P., Kwon H.J., Kosikova M., Ortega-Rodriguez U., Xiang R., Phue J.N. (2021). SARS-CoV-2 B.1.1.7 (Alpha) and B.1.351 (Beta) variants induce pathogenic patterns in K18-hACE2 transgenic mice distinct from early strains. Nat Commun.

[56] Zhou D., Dejnirattisai W., Supasa P., Liu C., Mentzer A.J., Ginn H.M. (2021). Evidence of escape of SARS-CoV-2 variant B.1.351 from natural and vaccine-induced sera. Cell.

[57] Tao K., Tzou P.L., Nouhin J., Gupta R.K., de Oliveira T., Kosakovsky Pond S.L. (2021). The biological and clinical significance of emerging SARS-CoV-2 variants. Nat Rev Genet.

[58] Zhang J., Cai Y., Xiao T., Lu J., Peng H., Sterling S.M. (2021). Structural impact on SARS-CoV-2 spike protein by D614G substitution. Science.

[59] Fujino T., Nomoto H., Kutsuna S., Ujiie M., Suzuki T., Sato R. (2021). Novel SARS-CoV-2 variant in travelers from Brazil to Japan. Emerg Infect Dis.

[60] Hirotsu Y., Omata M. (2021). Discovery of a SARS-CoV-2 variant from the P.1 lineage harboring K417T/E484K/N501Y mutations in Kofu, Japan. J Infect.

[61] Wang P., Casner R.G., Nair M.S., Wang M., Yu J., Cerutti G. (2021). Increased resistance of SARS-CoV-2 variant P.1 to antibody neutralization. Cell Host Microbe.

[62] Faria N.R., Mellan T.A., Whittaker C., Claro I.M., Candido D.D.S., Mishra S. (2021). Genomics and epidemiology of the P.1 SARS-CoV-2 lineage in Manaus, Brazil. Science.

[63] Tulimilli S.V., Dallavalasa S., Basavaraju C.G., Kumar Rao V., Chikkahonnaiah P., Madhunapantula S.V. (2022). Variants of severe acute respiratory syndrome coronavirus 2 (SARS-CoV-2) and vaccine effectiveness. Vaccines.

[64] Shiehzadegan S., Alaghemand N., Fox M., Venketaraman V. (2021). Analysis of the Delta variant B.1.617.2 COVID-19. Clin Pract.

[65] Dhawan M., Sharma A., Priyanka Thakur N., Rajkhowa T.K., Choudhary O.P. (2022). Delta variant (B.1.617.2) of SARS-CoV-2: mutations, impact, challenges and possible solutions. Hum Vaccines Immunother.

[66] Rahimi F., Talebi Bezmin Abadi A. (2022). Omicron: a highly transmissible SARS-CoV-2 variant. Gene Rep.

[67] Wang L., Cheng G. (2022). Sequence analysis of the emerging SARS-CoV-2 variant Omicron in South Africa. J Med Virol.

[68] Karim S.S.A., Karim Q.A. (2021). Omicron SARS-CoV-2 variant: a new chapter in the COVID-19 pandemic. Lancet.

[69] Ingraham N.E., Ingbar D.H. (2021). The Omicron variant of SARS-CoV-2: understanding the known and living with unknowns. Clin Transl Med.

[70] Liu W., Huang Z., Xiao J., Wu Y., Xia N., Yuan Q. (2024). Evolution of the SARS-CoV-2 omicron variants: genetic impact on viral fitness. Viruses.

[71] Yi B. (2025). Evaluation of the evolution of SARS-CoV-2 omicron variant and the spreading of LP. 8.1 and NB. 1.8. 1. medRxiv.

[72] Majumdar S., Sarkar R. (2022). Mutational and phylogenetic analyses of the two lineages of the Omicron variant. J Med Virol.

[73] Nutalai R., Zhou D., Tuekprakhon A., Ginn H.M., Supasa P., Liu C. (2022). Potent cross-reactive antibodies following Omicron breakthrough in vaccinees. Cell.

[74] Bazargan M., Elahi R., Esmaeilzadeh A. (2022). OMICRON: virology, immunopathogenesis, and laboratory diagnosis. J Gene Med.

[75] Hirotsu Y., Maejima M., Shibusawa M., Natori Y., Nagakubo Y., Hosaka K. (2022). Classification of Omicron BA.1, BA.1.1, and BA.2 sublineages by TaqMan assay consistent with whole genome analysis data. Int J Infect Dis.

[76] Yamasoba D., Kimura I., Nasser H., Morioka Y., Nao N., Ito J. (2022). Virological characteristics of the SARS-CoV-2 Omicron BA.2 spike. Cell.

[77] Aoki A., Adachi H., Mori Y., Ito M., Sato K., Kinoshita M. (2022). Rapid identification of SARS-CoV-2 Omicron BA.5 spike mutation F486V in clinical specimens using a high-resolution melting-based assay. Viruses.

[78] Parums D.V. (2023). Editorial: the XBB.1.5 (Kraken) subvariant of Omicron SARS-CoV-2 and its rapid global spread. Med Sci Monit.

[79] Naveed Siddiqui A., Musharaf I., Gulumbe B.H. (2025). The JN.1 variant of COVID-19: immune evasion, transmissibility, and implications for global health. Ther Adv Infect Dis.

[80] Chakraborty C., Bhattacharya M., Abdelhameed A.S. (2025). Recent SARS-CoV-2 evolution trajectories indicate the emergence of Omicron's several subvariants and the current rise of KP.3.1.1 and XEC. Virology.

[81] Branda F., Ciccozzi M., Scarpa F. (2024). Genetic variability of the recombinant SARS-CoV-2 XEC: is it a new evolutionary dead-end lineage?. New Microbes New Infect.

[82] Ataya F., Alamro A., Alghamdi A., Fouad D. (2025). SARS-CoV-2 spike mutations alter structure and energetics to modulate ACE2 binding immune evasion and viral adaptation. Sci Rep.

[83] Yao Z., Zhang L., Duan Y., Tang X., Lu J. (2024). Molecular insights into the adaptive evolution of SARS-CoV-2 spike protein. J Infect.

[84] Chen L., Zody M.C., Di Germanio C., Martinelli R., Mediavilla J.R., Cunningham M.H. (2021). Emergence of multiple SARS-CoV-2 antibody escape variants in an immunocompromised host undergoing convalescent plasma treatment. mSphere.

[85] Harvey W.T., Carabelli A.M., Jackson B., Gupta R.K., Thomson E.C., Harrison E.M. (2021). SARS-CoV-2 variants, spike mutations and immune escape. Nat Rev Microbiol.

[86] Tian F., Tong B., Sun L., Shi S., Zheng B., Wang Z. (2021). N501Y mutation of spike protein in SARS-CoV-2 strengthens its binding to receptor ACE2. eLife.

[87] Zhang L., Jackson C.B., Mou H., Ojha A., Peng H., Quinlan B.D. (2020). SARS-CoV-2 spike-protein D614G mutation increases virion spike density and infectivity. Nat Commun.

[88] Motozono C., Toyoda M., Zahradnik J., Saito A., Nasser H., Tan T.S. (2021). SARS-CoV-2 spike L452R variant evades cellular immunity and increases infectivity. Cell Host Microbe.

[89] Liu Y., Liu J., Johnson B.A., Xia H., Ku Z., Schindewolf C. (2022). Delta spike P681R mutation enhances SARS-CoV-2 fitness over Alpha variant. Cell Rep.

[90] Saito A., Irie T., Suzuki R., Maemura T., Nasser H., Uriu K. (2022). Enhanced fusogenicity and pathogenicity of SARS-CoV-2 Delta P681R mutation. Nature.

[91] Furusawa Y., Kiso M., Iida S., Uraki R., Hirata Y., Imai M. (2023). SARS-CoV-2 Delta variants, spike-P681R and D950N promote membrane fusion, spike-P681R enhances spike cleavage, but neither substitution affects pathogenicity in hamsters. EBioMedicine.

[92] Bannas P., Hambach J., Koch-Nolte F. (2017). Nanobodies and nanobody-based human heavy chain antibodies as antitumor therapeutics. Front Immunol.

[93] Shen Z., Sang Z., Shi Y. (2022). Nanobodies as a powerful platform for biomedicine. Trends Mol Med.

[94] Jin B.K., Odongo S., Radwanska M., Magez S. (2023). NANOBODIES®: a review of diagnostic and therapeutic applications. Int J Mol Sci.

[95] Muyldermans S. (2021). Applications of nanobodies. Ann Rev Anim Biosci.

[96] Collado Camps E., van Lith S.A.M., Frielink C., Lankhof J., Dijkgraaf I., Gotthardt M. (2021). CPPs to the test: effects on binding, uptake and biodistribution of a tumor targeting nanobody. Pharmaceuticals.

[97] Muyldermans S. (2013). Nanobodies: natural single-domain antibodies. Annu Rev Biochem.

[98] Harmand T.J., Islam A., Pishesha N., Ploegh H.L. (2021). Nanobodies as in vivo, non-invasive, imaging agents. RSC Chem Biol.

[99] de Beer M.A., Giepmans B.N.G. (2020). Nanobody-based probes for subcellular protein identification and visualization. Front Cell Neurosci.

[100] Alexander E., Leong K.W. (2024). Discovery of nanobodies: a comprehensive review of their applications and potential over the past five years. J Nanobiotechnol.

[101] Muyldermans S. (2021). A guide to: generation and design of nanobodies. FEBS J.

[102] Verma V., Sinha N., Raja A. (2025). Nanoscale warriors against viral invaders: a comprehensive review of nanobodies as potential antiviral therapeutics. mAbs.

[103] Mohammed A., Muustafa M.I. (2025). Nanobodies: a new frontier in antiviral therapies. SLAS Discov.

[104] Li H., Zhou Q., Cao N., Hu C., Wang J., He Y. (2025). Nanobodies and their derivatives: pioneering the future of cancer immunotherapy. Cell Commun Signal.

[105] Blueggel M., Gül D., Stauber R.H., Knauer S.K. (2025). Small but mighty: the versatility of nanobodies in modern medicine. Nanoscale Horiz.

[106] Duan Q., Ai T., Ma Y., Li R., Jin H., Chen X. (2025). Research progress on the application of neutralizing nanobodies in the prevention and treatment of viral infections. Microorganisms.

[107] Vijayandran K., Abdo A.I.K., Wong M.T.J., Balakrishnan V., Nordin F., Wan Kamarul (2025). Nanobodies targeting cytokines for the amelioration of autoimmune diseases. Int Immunopharmacol.

[108] Tanaka Y. (2023). Ozoralizumab: first nanobody® therapeutic for rheumatoid arthritis. Expert Opin Biol Ther.

[109] Wang R., Bai Z., Zhong W., Li C., Wang J., Xiang J. (2024). Synthesis, preclinical evaluation and pilot clinical translation of [^68^Ga]Ga-PMD22, a novel nanobody PET probe targeting CLDN18.2 of gastrointestinal cancer. Eur J Nucl Med Mol Imag.

[110] Zhang M., Chen D., Fu X., Meng H., Nan F., Sun Z. (2022). Autologous nanobody-derived fratricide-resistant CD7-CAR T-cell therapy for patients with relapsed and refractory T-cell acute lymphoblastic leukemia/lymphoma. Clin Cancer Res.

[111] Lu P., Zhang X., Yang J., Li J., Qiu L., Gong M. (2025). Nanobody-based naturally selected CD7-targeted CAR-T therapy for acute myeloid leukemia. Blood.

[112] Peyvandi F., Scully M., Kremer Hovinga J.A., Cataland S., Knöbl P., Wu H. (2016). Caplacizumab for acquired thrombotic thrombocytopenic purpura. N Engl J Med.

[113] Deiteren A., Krupka E., Bontinck L., Imberdis K., Conickx G., Bas S. (2025). A proof-of-mechanism trial in asthma with lunsekimig, a bispecific NANOBODY molecule. Eur Respir J.

[114] Xu P., Zhang Y., Guo J., Li H., Konrath S., Zhou P. (2024). A single-domain antibody targeting factor XII inhibits both thrombosis and inflammation. Nat Commun.

[115] Ye G., Pan R., Bu F., Zheng J., Mendoza A., Wen W. (2023). Discovery of Nanosota-2, -3, and -4 as super potent and broad-spectrum therapeutic nanobody candidates against COVID-19. J Virol.

[116] Harmsen M.M., Ackerschott B., de Smit H. (2024). Serum immunoglobulin or albumin binding single-domain antibodies that enable tailored half-life extension of biologics in multiple animal species. Front Immunol.

[117] Vaskuri G.J., Ye G., Bu F., Yang D., Jonsson C.B., Turner-Hubbard H. (2025). Long-circulating nanobody confers durable prophylaxis against severe acute respiratory syndrome coronavirus 2 omicron infection. Adv Nanobiomed Res.

[118] Mo X., Xu Y., Han F., Wang Y., Zhou M., He C. (2025). Engineered B7-H3-targeted VHH-Fc fusion antibody demonstrates rapid tumor accumulation for infrared imaging in pancreatic cancer xenograft model. Bioorg Med Chem.

[119] Xiang Y., Xu J., McGovern B.L., Ranzenigo A., Huang W., Sang Z. (2024). Adaptive multi-epitope targeting and avidity-enhanced nanobody platform for ultrapotent, durable antiviral therapy. Cell.

[120] Zhang M., Qin L., Guo R., Li J., Huang S., Sheng C. (2025). A novel bispecific nanobody protects mice against RSV infection via intranasal administration. J Virol.

[121] Matthys A., Felix J., Catani J.P.P., Roose K., Nerinckx W., Van Buyten B. (2025). Single-domain antibodies directed against hemagglutinin and neuraminidase protect against influenza B viruses. Nat Commun.

[122] Feng X., Wang H. (2023). Emerging landscape of nanobodies and their neutralizing applications against SARS-CoV-2 virus. ACS Pharmacol Transl Sci.

[123] Najmeddin A., Bahrololoumi Shapourabadi M., Behdani M., Dorkoosh F. (2021). Nanobodies as powerful pulmonary targeted biotherapeutics against SARS-CoV-2, pharmaceutical point of view. Biochim Biophys Acta Gen Subj.

[124] Weinstein J.B., Bates T.A., Leier H.C., McBride S.K., Barklis E., Tafesse F.G. (2022). A potent alpaca-derived nanobody that neutralizes SARS-CoV-2 variants. iScience.

[125] Hong J., Kwon H.J., Cachau R., Chen C.Z., Butay K.J., Duan Z. (2022). Dromedary camel nanobodies broadly neutralize SARS-CoV-2 variants. Proc Natl Acad Sci U S A.

[126] Winkler E.S., Bailey A.L., Kafai N.M., Nair S., McCune B.T., Yu J. (2020). SARS-CoV-2 infection of human ACE2-transgenic mice causes severe lung inflammation and impaired function. Nat Immunol.

[127] Rosenfeld R., Noy-Porat T., Mechaly A., Makdasi E., Levy Y., Alcalay R. (2021). Post-exposure protection of SARS-CoV-2 lethal infected K18-hACE2 transgenic mice by neutralizing human monoclonal antibody. Nat Commun.

[128] Guo Y., Zhang G., Yang Q., Xie X., Lu Y., Cheng X. (2023). Discovery and characterization of potent pan-variant SARS-CoV-2 neutralizing antibodies from individuals with omicron breakthrough infection. Nat Commun.

[129] Chen R.E., Winkler E.S., Case J.B., Aziati I.D., Bricker T.L., Joshi A. (2021). *In vivo* monoclonal antibody efficacy against SARS-CoV-2 variant strains. Nature.

[130] Solodkov P.P., Najakshin A.M., Chikaev N.A., Kulemzin S.V., Mechetina L.V., Baranov K.O. (2024). Serial llama immunization with various SARS-CoV-2 RBD variants induces broad spectrum virus-neutralizing nanobodies. Vaccines.

[131] Chen W.H., Hajduczki A., Martinez E.J., Bai H., Matz H., Hill T.M. (2023). Shark nanobodies with potent SARS-CoV-2 neutralizing activity and broad sarbecovirus reactivity. Nat Commun.

[132] Walter J.D., Scherer M., Hutter C.A.J., Garaeva A.A., Zimmermann I., Wyss M. (2022). Biparatopic sybodies neutralize SARS-CoV-2 variants of concern and mitigate drug resistance. EMBO Rep.

[133] Fu Y., da Fonseca Rezende E.Mello J., Fleming B.D., Renn A., Chen C.Z., Hu X. (2022). A humanized nanobody phage display library yields potent binders of SARS CoV-2 spike. PLoS One.

[134] Xu J., Xu K., Jung S., Conte A., Lieberman J., Muecksch F. (2021). Nanobodies from camelid mice and llamas neutralize SARS-CoV-2 variants. Nature.

[135] Ye G., Bu F., Saxena D., Turner-Hubbard H., Herbst M., Spiller B. (2024). Discovery of Nanosota-9 as Anti-Omicron nanobody therapeutic candidate. PLoS Pathog.

[136] Wang J., Shi B., Chen H., Yu M., Wang P., Qian Z. (2024). Engineered multivalent nanobodies efficiently neutralize SARS-CoV-2 Omicron subvariants BA.1, BA.4/5, XBB.1 and BQ.1.1. Vaccines.

[137] Cornish K., Huo J., Jones L., Sharma P., Thrush J.W., Abdelkarim S. (2024). Structural and functional characterization of nanobodies that neutralize Omicron variants of SARS-CoV-2. Open Biol.

[138] He L., Wu Q., Zhang Z., Chen L., Yu K., Li L. (2024). Development of broad-spectrum nanobodies for the therapy and diagnosis of SARS-CoV-2 and its multiple variants. Mol Pharm.

[139] Zheng L., Wang H., Liu X., Xu C., Tian M., Shi G. (2024). A panel of multivalent nanobodies broadly neutralizing Omicron subvariants and recombinant. J Med Virol.

[140] Aksu M., Kumar P., Güttler T., Taxer W., Gregor K., Mußil B. (2024). Nanobodies to multiple spike variants and inhalation of nanobody-containing aerosols neutralize SARS-CoV-2 in cell culture and hamsters. Antivir Res.

[141] Yao H., Wang H., Zhang Z., Lu Y., Zhang Z., Zhang Y. (2023). A potent and broad-spectrum neutralizing nanobody for SARS-CoV-2 viruses, including all major Omicron strains. MedComm (2020).

[142] Dong H., Zhou R., Chen J., Wei J., Wei Z., Yang Z. (2024). Super broad and protective nanobodies against sarbecoviruses including SARS-CoV-1 and the divergent SARS-CoV-2 subvariant KP.3.1.1. PLoS Pathog.

[143] Liu B., Liu H., Han P., Wang X., Wang C., Yan X. (2024). Enhanced potency of an IgM-like nanobody targeting conserved epitope in SARS-CoV-2 spike N-terminal domain. Signal Transduct Targeted Ther.

[144] Rossotti M.A., van Faassen H., Tran A.T., Sheff J., Sandhu J.K., Duque D. (2022). Arsenal of nanobodies shows broad-spectrum neutralization against SARS-CoV-2 variants of concern in vitro and in vivo in hamster models. Commun Biol.

[145] Wang X., Xie Y., Liu H., Lei W., Xu K., Wu L. (2023). A broadly neutralizing nanobody targeting the highly conserved S2 subunit of sarbecoviruses. Sci Bull.

[146] Feng B., Li C., Zhang Z., Huang Y., Liu B., Zhang Z. (2025). A shark-derived broadly neutralizing nanobody targeting a highly conserved epitope on the S2 domain of sarbecoviruses. J Nanobiotechnol.

[147] Buffington J., Duan Z., Kwon H.J., Hong J., Li D., Feng M. (2023). Identification of nurse shark V_NAR_ single-domain antibodies targeting the spike S2 subunit of SARS-CoV-2. FASEB J.

[148] Mast F.D., Fridy P.C., Ketaren N.E., Wang J., Jacobs E.Y., Olivier J.P. (2021). Highly synergistic combinations of nanobodies that target SARS-CoV-2 and are resistant to escape. eLife.

[149] Venit T., Blavier J., Maseko S.B., Shu S., Espada L., Breunig C. (2024). Nanobody against SARS-CoV-2 non-structural protein Nsp9 inhibits viral replication in human airway epithelia. Mol Ther Nucleic Acids.

[150] Segovia-de Los Santos P., Padula-Roca C., Simon X., Echaides C., Lassabe G., Gonzalez-Sapienza G. (2023). A highly sensitive nanobody-based immunoassay detecting SARS-CoV-2 nucleocapsid protein using all-recombinant reagents. Front Immunol.

[151] Yamamoto Y., Noguchi K. (2025). Structural insights into the SARS-CoV-2 spike protein and its implications for antibody resistance. Biomolecules.

[152] Chi X., Zhang X., Pan S., Yu Y., Shi Y., Lin T. (2022). An ultrapotent RBD-targeted biparatopic nanobody neutralizes broad SARS-CoV-2 variants. Signal Transduct Targeted Ther.

[153] Wagner T.R., Schnepf D., Beer J., Ruetalo N., Klingel K., Kaiser P.D. (2022). Biparatopic nanobodies protect mice from lethal challenge with SARS-CoV-2 variants of concern. EMBO Rep.

[154] Favorskaya I.A., Shcheblyakov D.V., Esmagambetov I.B., Dolzhikova I.V., Alekseeva I.A., Korobkova A.I. (2022). Single-domain antibodies efficiently neutralize SARS-CoV-2 variants of concern. Front Immunol.

[155] Zupancic J.M., Schardt J.S., Desai A.A., Makowski E.K., Smith M.D., Pornnoppadol G. (2021). Engineered multivalent nanobodies potently and broadly neutralize SARS-CoV-2 variants. Adv Ther.

[156] Ma H., Zhang X., Zheng P., Dube P.H., Zeng W., Chen S. (2022). Hetero-bivalent nanobodies provide broad-spectrum protection against SARS-CoV-2 variants of concern including Omicron. Cell Res.

[157] Wu X., Cheng L., Fu M., Huang B., Zhu L., Xu S. (2021). A potent bispecific nanobody protects hACE2 mice against SARS-CoV-2 infection via intranasal administration. Cell Rep.

[158] Wu Y. (2025). Trends in nanobody technology in industrialization. Discov Nano.

[159] Duggan S. (2018). Caplacizumab: first global approval. Drugs.

[160] Wang R., Wang Z., Zhang H., Liu X., Zhu P., Zhou F. (2026). Modern strategies in nanobody engineering and functionalization: from discovery to biosensing application. TrAC Trends Anal Chem.

[161] Rossotti M.A., Bélanger K., Henry K.A., Tanha J. (2022). Immunogenicity and humanization of single-domain antibodies. FEBS J.

[162] Mei Y., Chen Y., Sivaccumar J.P., An Z., Xia N., Luo W. (2022). Research progress and applications of nanobody in human infectious diseases. Front Pharmacol.

